# Sex-specific electrophysiological and behavioral responses to nitrous oxide in a murine model of neuropathic pain

**DOI:** 10.1186/s13293-026-00945-2

**Published:** 2026-07-07

**Authors:** Gemma González-Hernández, Sonia Torres-Sanchez, Daniel Sanchez-Morillo, Tomi Rantamäki, Esther Berrocoso

**Affiliations:** 1 Laboratory of Neurotherapeutics, Drug Research Program, Division of Pharmacology and Pharmacotherapy, Faculty of Pharmacy, University of Helsinki, Helsinki, Finland; 2 SleepWell Research Program, Faculty of Medicine, University of Helsinki, Helsinki, Finland; 3 Neuropsychopharmacology and Psychobiology Research Group, Department of Neuroscience, Faculty of Medicine, University of Cádiz, Cádiz, 11003 Spain; 4 Centro de Investigación Biomédica en Red de Salud Mental (CIBERSAM), Instituto de Salud Carlos III, Madrid, 28029 Spain; 5 Instituto de Investigación e Innovación Biomédica de Cádiz (INiBICA), Hospital Universitario Puerta del Mar, Cádiz, 11009 Spain; 6 Department of Automation Engineering, Electronics and Computer Architecture and Networks, School of Engineering, University of Cadiz, Cádiz, 11519 Spain

**Keywords:** Chronic pain, Neuropathic pain, Depression, Rapid-acting antidepressant, Nitrous oxide, EEG

## Abstract

Chronic pain–depression comorbidity is characterized by sensory hypersensitivity and emotional dysregulation, and has been linked to alterations in prefrontal network connectivity. It represents a major therapeutic challenge due to its pronounced sex differences in symptom manifestation and increased treatment resistance. Nitrous oxide (N₂O), an inhalational anesthetic traditionally used for its analgesic properties, has recently emerged as a potential rapid-acting antidepressant, with evidence suggesting both rapid and sustained antidepressant effects. Here, we evaluated the behavioral and electrophysiological effects of N₂O (50%, 1 h) in adult male and female mice subjected to the chronic constriction injury (CCI) model of neuropathic pain, assessing its potential to simultaneously target nociceptive and affective symptoms, as well as locomotor behavior. N₂O reduced locomotor activity in both sexes, with longer-lasting effects in males. EEG recordings revealed decreased low-frequency spectral power in both sham and CCI animals, with more widespread effects in females. In the acetone test, N₂O produced analgesia in CCI males but no significant effect in females, while stimulus application increased oscillatory EEG activity across groups and sexes. Notably, CCI females showed an attenuated electrophysiological response compared with Sham-Air controls. In sham animals, N₂O reduced post-stimulus power, whereas in CCI mice it restored prefrontal EEG activity, enhancing the electrophysiological responses in both sexes. In the tail suspension test, only CCI males exhibited a prodepressive phenotype, while no antidepressant-like effects were observed in either sex 24 h post-treatment. These findings demonstrate sex-specific behavioral and neurophysiological responses to N₂O, highlighting the importance of incorporating sex as a biological variable in NMDA receptor–targeted interventions.

**Supplementary Information:**

The online version contains supplementary material available at https://doi.org/10.1186/s13293-026-00945-2.

## Abstract (plain language summary)

Chronic pain and depression often occur together and can severely affect quality of life. People living with both conditions frequently experience increased pain sensitivity, low mood, reduced motivation, and poor response to treatment. Importantly, men and women can experience these symptoms differently, yet many studies do not adequately investigate these sex differences. Nitrous oxide (N₂O), commonly known as “laughing gas,” is used in clinical practice for pain relief and has recently attracted interest as a potential fast-acting antidepressant. However, its effects in chronic pain–related depression, as well as potential differences between males and females, have not been explored. In this study, we investigated how N₂O affects pain- and mood-related behaviors and brain activity in male and female mice with nerve injury–induced chronic pain. After N₂O exposure, we evaluated movement activity, pain sensitivity, depressive-like behavior, and electroencephalographic (EEG) activity. Our findings reveal that N₂O produced distinct behavioral and EEG responses in male and female mice. Particularly, N₂O similarly reduced movement activity and altered EEG activity in both sexes, although the timing and magnitude of these effects differed between sexes. N₂O reduced pain sensitivity in male mice with chronic pain, but not in females, and did not produce clear antidepressant-like effects 24 h after treatment in either sex. Overall, our findings show that males and females respond differently to N₂O treatment and highlight the importance of considering sex differences when developing new treatments for chronic pain and depression.

**Supplementary Information:**

The online version contains supplementary material available at https://doi.org/10.1186/s13293-026-00945-2.

## Highlights

N_2_O elicits distinct behavioral and EEG responses in male and female mice.

N_2_O induces a prominent locomotor suppression after administration that is longer-lasting in males.

N_2_O produces analgesia in males but no significant effects in females.

N_2_O reduces prefrontal low-frequency EEG power during the first hour post-treatment.

N_2_O restores prefrontal EEG activity of CCI mice in response to a sensory stimulus.

**Supplementary Information:**

The online version contains supplementary material available at https://doi.org/10.1186/s13293-026-00945-2.

## Introduction

Chronic pain is a physically and psychologically debilitating condition and represents one of the most challenging forms of pain in terms of symptom burden, associated comorbidities, and treatment [1, 2]. Chronic pain is accompanied by substantial psychological distress and a high degree of disability, as reflected by the elevated prevalence of psychopathologies and comorbid conditions [3]. Notably, there is a high rate of comorbidity between chronic pain and depression, such that chronic pain represents a major risk factor for depression, while depression can exacerbate pain symptoms and hinder treatment efficacy [4, 5]. Patients suffering from chronic pain–depression comorbidity constitute one of the most vulnerable populations, exhibiting more severe disability, poorer prognosis, and frequent treatment resistance [6, 7]. However, the mechanisms underlying this comorbidity remain poorly understood, and therapeutic options are limited.

Nitrous oxide (N₂O) is an inhalational anesthetic with a long history of safe clinical use as an add-on to general anesthesia, obstetric analgesia (e.g. labor), and sedation for short painful procedures (e.g. dentistry) [8]. In recent years, several clinical trials have suggested that N₂O may also exert rapid and durable antidepressant effects in patients with major depressive disorder and treatment-resistant major depression [9–12]. Although clinical and preclinical studies investigating N₂O remain limited, its dual analgesic and rapid antidepressant actions suggest it may represent a promising therapeutic option for patients suffering from comorbid chronic pain and depression. N₂O acts through multifaceted and not fully elucidated mechanisms of action. It is primarily a non-competitive antagonist of N-methyl-D-aspartate receptors (NMDARs) and also weakly inhibits AMPA (α-amino-3-hydroxy-5-methyl-4-isoxazolepropionic acid) glutamate receptors while modestly potentiating GABA_A_ (gamma-aminobutyric acid subtype A) receptors, among other ion channel–mediated actions [13]. Additionally, it can activate α-adrenergic and opioid receptors. N₂O is known to provide acute analgesic effects primarily through opioid-mediated pathways, whereas sustained effects on chronic pain likely involve NMDAR blockade and inhibition of glutamate-driven central sensitization [14, 15]. However, the underlying mechanisms of its antidepressant effects remain largely unknown.

Reflecting these widespread actions on neurotransmission, N₂O has been shown to alter cortical electroencephalographic (EEG) activity and brain functional connectivity in networks contributing to cognition and emotion [13], including the default mode network. Within this network, the medial prefrontal cortex (mPFC) represents a critical hub in pain and mood-related networks, integrating cognitive, affective, and sensory information and modulating these processes through its projections to diverse brain regions and the spinal cord [16]. Despite limited evidence, recent human studies show connectivity changes that persist at least 24 h following a single administration of N₂O [17–19].

The present study evaluated the potential of N_2_O to target both nociceptive and affective symptoms in a murine model of pain-induced depression [20]. We hypothesized that the comorbidity model would produce behavioral and electrophysiological alterations that could be ameliorated by N₂O treatment. Specifically, we examined the effects of a 1-hour inhalation of 50% N₂O on locomotor behavior and mPFC EEG activity, as well as on thermal hypersensitivity (acetone test) and behavioral despair (tail suspension test; TST).

## Materials and methods

### Animals

Male and female adult C57BL/6J mice (weighting 24–32 g males and 20–25 g females) were used in this study and maintained group-housed in separate rooms under standard laboratory conditions (22 °C, 12-h light–dark cycle, lights on at 6:30a.m ~ Zeitgeber Time 0; ZT0, food and water ad libitum). All procedures were approved by the Committee for Animal Experimentation at the University of Cadiz, conforming to the European Commission’s Directive (2010/63/EU) and Spanish Law (RD 53/2013) regulating animal research.

### Experimental design

**Fig. 1:**
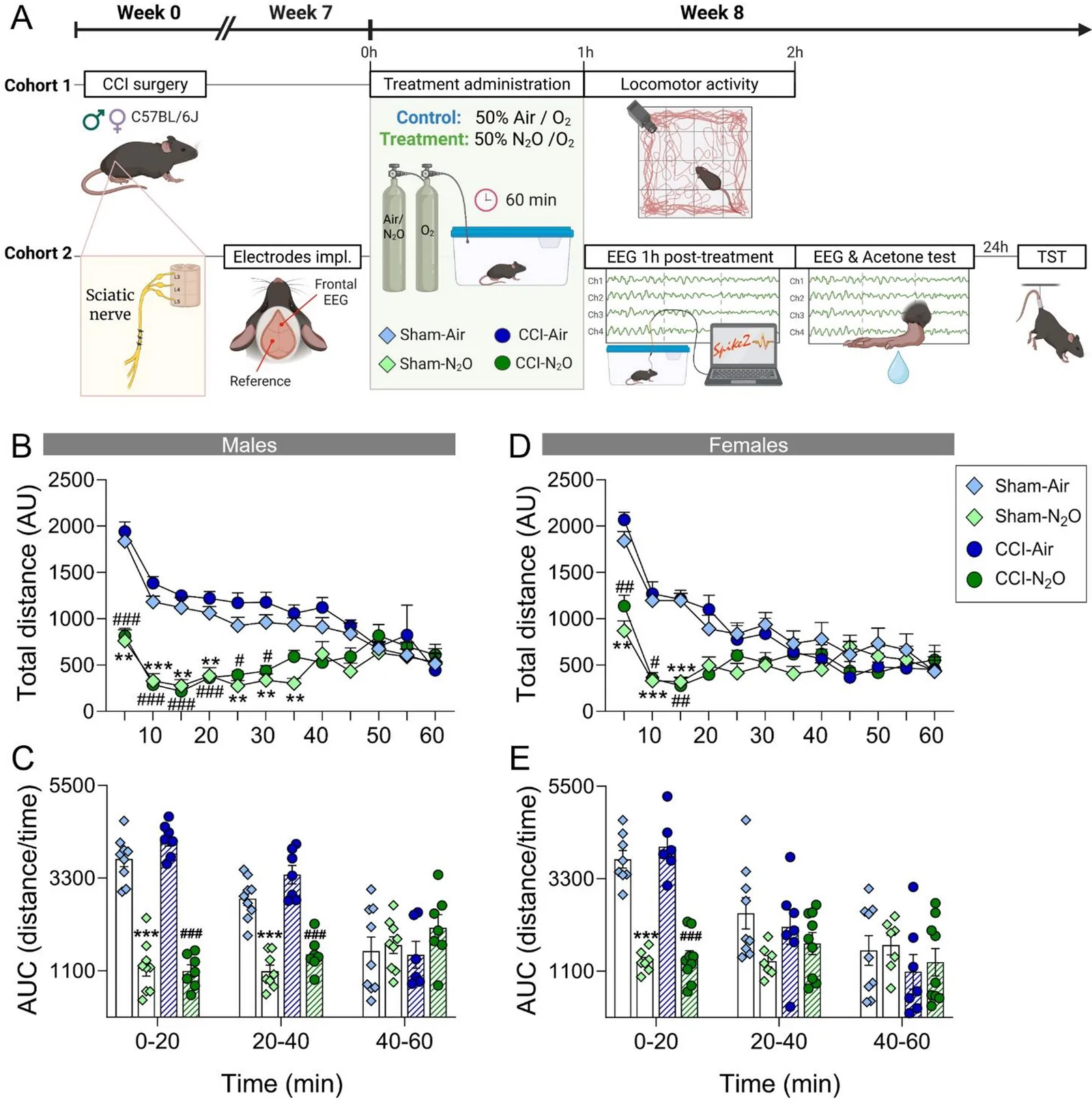
Effects of N_2_O treatment on spontaneous locomotor activity in male and female mice. **(A)** Schematic representation of the experimental design. Time course of total distance traveled by male **(B)** and female **(D)** mice during the 1-hour post-treatment interval. AUC calculated for consecutive 20-minute intervals of total distance traveled by male **(C)** and female **(E)** mice. Data are presented as mean ± SEM (*n* = 7–9 per group). *p < 0.05, **p < 0.01, ***p < 0.001 vs. Sham-Air; ^#^*p* < 0.05, ^##^*p* < 0.01, ^###^*p* < 0.001 vs. CCI-Air; RM ANOVA or mixed-effects models (REML) followed by Tukey’s *post hoc* test. Detailed statistical results are provided in Table S1

The experimental design is illustrated in Fig. 1A. Eight-week-old mice underwent chronic constriction injury (CCI) of the sciatic nerve or sham surgery. Nociceptive and affective phenotypes were allowed to develop over the following seven weeks. At week 8 post-surgery, animals received a 50% N_2_O/O_2_ treatment for 1 h and were randomly assigned, within each sex, to one of four groups: Sham-Air, Sham-N₂O, CCI-Air or CCI-N₂O. Male and female mice were studied using the same experimental protocol but in separate, consecutive cohorts rather than in parallel, and therefore sex was not included as an experimental factor for direct statistical comparisons. Cohort 1 was used to assess locomotor activity during the 1-hour post-treatment period. In Cohort 2, animals were implanted with EEG electrodes at week 7 post-surgery and allowed one week for recovery before treatment. Prefrontal EEG activity was recorded during the 1-hour post-treatment interval, after which nociceptive behavior was assessed using the acetone test with simultaneous EEG recording. Twenty-four hours after treatment administration, mice underwent the tail suspension test to evaluate behavioral despair. A small independent group of male mice was used to assess the sustained effects of N_2_O on nociception in the acetone test at 6 h and 24 h following treatment administration (Supplementary Information File, Fig. S7).

Some samples were missing or excluded based on predefined technical criteria, including incomplete treatment delivery, loss or damage of electrode connection, poor EEG signal quality, or technical issues encountered during the combined EEG-behavioral protocol. As these issues primarily affected EEG recordings, sample sizes were reduced for EEG analyses compared to the behavioral datasets. Final sample sizes for each experimental group and dataset are reported in the corresponding figure legends.

### Chronic neuropathic pain model

CCI was used as a model of neuropathic pain, as it recapitulates pain symptomatology observed in patients and allows the study of depressive-like behaviors associated with chronic pain, which emerge approximately 7–11 weeks after nerve injury in mice [21, 22]. The procedure was performed as previously described [21]. Briefly, mice were anesthetized with 4% isoflurane for induction and maintained on 1.5-2% isoflurane. After shaving and disinfecting the surgical area, a skin incision was made on the lateral surface of the right hind thigh, and the musculature dissected to expose the sciatic nerve proximal to its trifurcation. Three chromic gut ligatures (6 − 0) were loosely tied around the nerve at intervals of 1.0 mm, ensuring that blood flow was not interrupted and the skin incision was subsequently closed with 4 − 0 silk sutures. Sham-operated mice underwent the same procedure, except for nerve ligation.

### Stereotaxic surgery for EEG electrodes implantation

Seven weeks after CCI induction, animals underwent stereotaxic surgery for the implantation of EEG recording electrodes (Cohort 2). Briefly, mice were anesthetized with isoflurane (4% for induction and 2-2.5% for maintenance) and placed in a stereotaxic frame. Local anesthetics were subcutaneously (s.c.) administered (lidocaine, 3.5 mg/kg; bupivacaine, 2 mg/kg), after which the surgical area was shaved and disinfected, and a midline scalp incision was made. Following removal of the periosteum and exposure of the skull, two burr holes were drilled using a steel-bit electric microdrill at the coordinates selected for recording, as determined using the Franklin and Paxinos mouse brain atlas [23]. For fronto-parietal derivation, a recording electrode over the mPFC (AP: +1.9 mm, ML: −0.5 mm) and a parietal reference electrode (AP: −2.9 mm, ML: +2.5 mm) were implanted. Electrodes were inserted into the burr holes and positioned epidurally over the cortex. Electrodes consisted of two stainless steel screws (0.86 mm diameter; 19010-10, Fine Science Tools) soldered to a lightweight electronic connector (Mill-Max 852-10-100-10-001000) using a PFA-coated silver wire (AM Systems, Catalog No 785500), allowing connection to the recording cable connector (Mill-Max 853-43-100-10-001000). Finally, the entire assembly was secured with dental cement, the skin was sutured around the formed head cap, and physiological saline was administered s.c. to support recovery before animals were returned to group housing.

### Nitrous oxide administration

N_2_O was administered as a gas mixture containing 50% N_2_O and 50% oxygen (O_2_) for 60 min, as previously described in clinical [11] and preclinical studies [24]. Briefly, N_2_O and O_2_ (Alphagaz™ 1 N_2_O - P0311L50R0A001 and O_2_ - I1001L50R2A001; Air Liquide, Spain) were delivered *via* a tubing system into custom-made administration chambers (16 × 25 × 13,5 cm), where gases were mixed at a 1:1 ratio at a final flow rate of 5–7 L/min. Using an identical gas delivery setup, control animals received a sham treatment consisting of compressed breathable air (I4550L50R2A001, Air Liquide, Spain) mixed with O_2_ to match the 50% O_2_ concentration of the N_2_O-treated group. All treatment administration sessions took place during the light phase, between ZT3-6 (ZT, Zeitgeber Time).

### Locomotor activity recordings

The effect of N_2_O on locomotor activity was assessed during the 1-hour post-treatment period (ZT4-7; Cohort 1). Immediately after treatment, mice were gently removed from the administration chamber and individually placed in empty home-cages with transparent lids to allow video recording. Locomotor activity was recorded for 1 h and subsequently analyzed using SMART video-tracking software (Panlab, S.L., Barcelona, Spain). The total distance traveled was quantified in 5-minute time bins across the full recording period to evaluate the temporal dynamics of N₂O-induced changes in locomotor activity. In addition, area under the curve (AUC) values were calculated for consecutive 20-minute intervals of total distance traveled.

### EEG recordings

Electrophysiological recordings were performed in awake, freely moving mice during the 1-hour post-treatment interval (ZT4-7; Cohort 2). The habituation to the recording setup consisted in two 30-minute habituation sessions on the days preceding the experiment. In addition, mice were acclimated to the recording room for at least 30 min prior to baseline (pre-treatment) EEG acquisition using a multichannel electrophysiology system. The EEG signal was amplified (gain 10 K) and band-pass filtered (0.3–100 Hz) with a notch filter using a 64 AC amplifier (Cibertec, Spain). Oscillatory activity was digitized at a sampling rate of 25 kHz using CED Micro1401 and Spike2 computer software (Cambridge Electronic Design, Cambridge, UK).

### Nociceptive and depressive-like behavioral assessment

**Acetone test** Cold hypersensitivity was assessed using the acetone evaporation test immediately after the 1-hour EEG recordings. Mice, still connected to the EEG setup, were carefully transferred to individual plastic chambers placed on an elevated mesh floor and allowed to habituate for several minutes to normalize the EEG signal. A 50 µL drop of acetone was applied to the plantar surface of the CCI-injured hind paw (ipsilateral) to induce evaporative cooling. The duration of hindlimb lifting, licking, and/or shaking was recorded for 60 s, and the time of response was calculated as the average of three acetone applications [25, 26]. All acetone test sessions were conducted within ZT5–8. After testing, mice were returned to their home cages and left undisturbed until the following morning.

#### Tail suspension test (TST)

24 h after treatment, the TST was used to evaluate depressive-like behavior. Animals were habituated to the behavioral room 30–60 min prior to testing. Mice were suspended, 20 cm above the floor, by the distal end of the tail using adhesive tape for a 6-minute recorded test session [27]. Behavior was manually scored to determine the total time spent immobile, defined as hanging passively without performing any active movements. All TST sessions were conducted within ZT3-6.

### EEG data analysis

Electrophysiological recordings were analyzed offline using Spike2 software (Cambridge Electronic Devices, UK) and MATLAB (MathWorks). Prior to analysis, signals were downsampled to 1250 Hz and filtered to remove frequencies below 1 Hz (denoising). Spectral power was computed for the following frequency bands: delta (1–4 Hz), theta (4–8 Hz), alpha (8–12 Hz), beta (12–30 Hz), gamma low (30–45 Hz), and gamma high (60–100 Hz), using the Chronux toolbox (http://chronux.org) in MATLAB.

For the 1-hour post-treatment EEG recordings, spectral analysis was performed using a Short-Time Fourier Transform (STFT) with a frequency resolution of 0.0095 Hz, applied to consecutive 60-second epochs across the entire recording. For EEG recordings obtained during the acetone test, higher temporal resolution was required to capture rapid stimulus-evoked changes. Therefore, STFT analysis was performed on 1-second epochs across a 60-second window (30s pre-acetone and 30s post-acetone). These time-frequency representations were calculated for each trial as well as the mean of the three acetone trials. Zero-padding was applied prior to the STFT to improve frequency resolution (0.15 Hz). All signals were normalized to the median spectral power of the baseline EEG recording for each individual mouse.

### Statistical analysis

Data are presented as Mean ± Standard error of the mean (SEM). Statistical analyses were performed using ordinary or repeated-measures ANOVA, or mixed-effects models (REML), followed by Tukey’s multiple-comparisons test, as appropriate. For the EEG-acetone data, Uncorrected Fisher’s LSD *post hoc* analysis was applied. For pairwise comparisons, Student’s two-tailed unpaired *t*-test was used for normally distributed data, whereas the Mann–Whitney *U* test was applied to non-normally distributed data. Statistical significance was set at *p* < 0.05 for all analyses. Prior to statistical testing, outliers were identified using Grubb’s test (α = 0.05), and data normality was assessed using the Shapiro–Wilk test. All statistical analyses and graphical representations were performed using GraphPad Prism v.10 (GraphPad Software, San Diego, CA, USA). Specific statistical tests applied for each dataset are indicated in the corresponding figure legends and detailed statistical results are provided in Supplementary Tables S1–S6. Direct statistical comparisons between male and female rodents were not performed because experiments in each sex were conducted in separate, consecutive cohorts of animals rather than in parallel, precluding the use of sex as an experimental factor.

## Results

### Effects of N₂O treatment on spontaneous locomotor activity in male and female mice

Given the previously reported effects of N₂O on locomotor activity in male mice [24], we examined the temporal dynamics of N₂O-induced changes during the first hour post-treatment (Fig. 1; Supplementary Table S1).

In males, analysis of the time course of total distance traveled (Fig. 1B) revealed a significant interaction between time and treatment (Time x Treatment: *p* < 0.0001), with no main effect of the model. Sham-Air and CCI-Air mice did not differ in total distance traveled and displayed only the expected gradual decline in locomotor activity over time as exploratory behavior decreased. In contrast, N₂O induced a pronounced decrease in total distance traveled in both sham (Sham-Air vs. Sham-N₂O; 5 min: *p* < 0.01, 10 min: *p* < 0.001, 15–35 min: *p* < 0.01; Fig. 1B) and CCI animals (CCI-Air vs. CCI-N₂O; 5–20 min: *p* < 0.001, 25–30 min: *p* < 0.05; Fig. 1B). These findings were corroborated by area under the curve (AUC) analyses (Fig. 1C), in which a significant interaction between time and treatment was again detected (Time x Treatment: *p* < 0.0001). Male mice exhibited significantly reduced locomotor activity in the Sham-N₂O group compared with Sham-Air, and in the CCI-N₂O group compared with CCI-Air, during both the 0–20 min interval (*p* < 0.001 vs. Sham-Air and CCI-Air; Fig. 1C) and the 20–40 min interval (*p* < 0.001 vs. Sham-Air and CCI-Air; Fig. 1C). These differences were no longer observed during the final 40–60 min interval.

In female mice, a significant interaction between time and treatment was similarly detected in the analysis of the time course of total distance traveled (Time x Treatment: *p* < 0.0001). Additionally, a Time x Model interaction was also identified (*p* < 0.05). N₂O administration significantly reduced total distance traveled, but this effect was transient, lasting only up to 15 min following gas withdrawal in both sham (Sham-Air vs. Sham-N₂O; 5 min: *p* < 0.01, 10–15 min: *p* < 0.001; Fig. 1D), and CCI mice (CCI-Air vs. CCI-N₂O; 5 min: *p* < 0.01, 10 min: *p* < 0.05, 15 min: *p* < 0.01; Fig. 1D). The AUC analyses also revealed a significant interaction between time and treatment (Time x Treatment: *p* < 0.0001), with the significant effects restricted to the initial 0–20 min interval (*p* < 0.001 vs. Sham-Air and CCI-Air; Fig. 1E). No group differences were detected during the subsequent 20–40 min or 40–60 min intervals.

### Effects of N₂O treatment on EEG activity in the prefrontal cortex of male and female mice

**Fig. 2:**
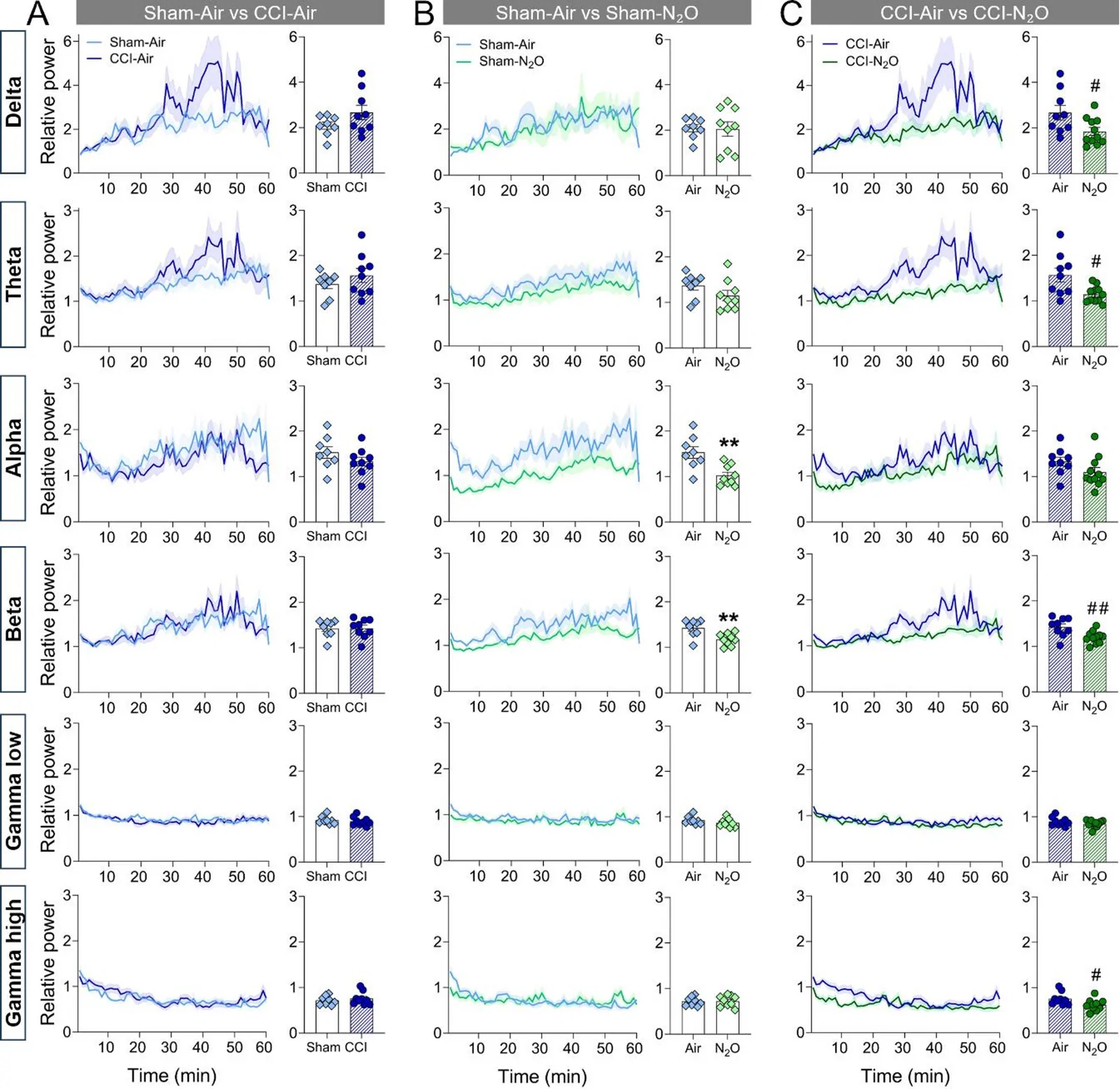
Impact of the CCI model and N₂O treatment on prefrontal EEG activity during the 1-hour post-treatment period in male mice. EEG spectral power recorded across the 1-hour post-treatment period for comparisons between **(A)** Sham-Air and CCI-Air groups (*n* = 8–9), **(B)** Sham-Air and Sham-N_2_O groups (*n* = 8–9), and **(C)** CCI-Air and CCI-N_2_O groups (*n* = 9–11). EEG oscillatory activity is represented as time-course relative power (normalized to baseline), together with comparisons of the average total power for each frequency band: delta (1–4 Hz), theta (4–8 Hz), alpha (8–12 Hz), beta (12–30 Hz), gamma low (30–45 Hz), and gamma high (60–100 Hz). Data are presented as mean ± SEM. **p < 0.01 vs. Sham-Air; ^#^*p* < 0.05, ^##^*p* < 0.01 vs. CCI-Air; Student’s two-tailed unpaired *t* test. Detailed statistical results are provided in Table S2

**Fig. 3:**
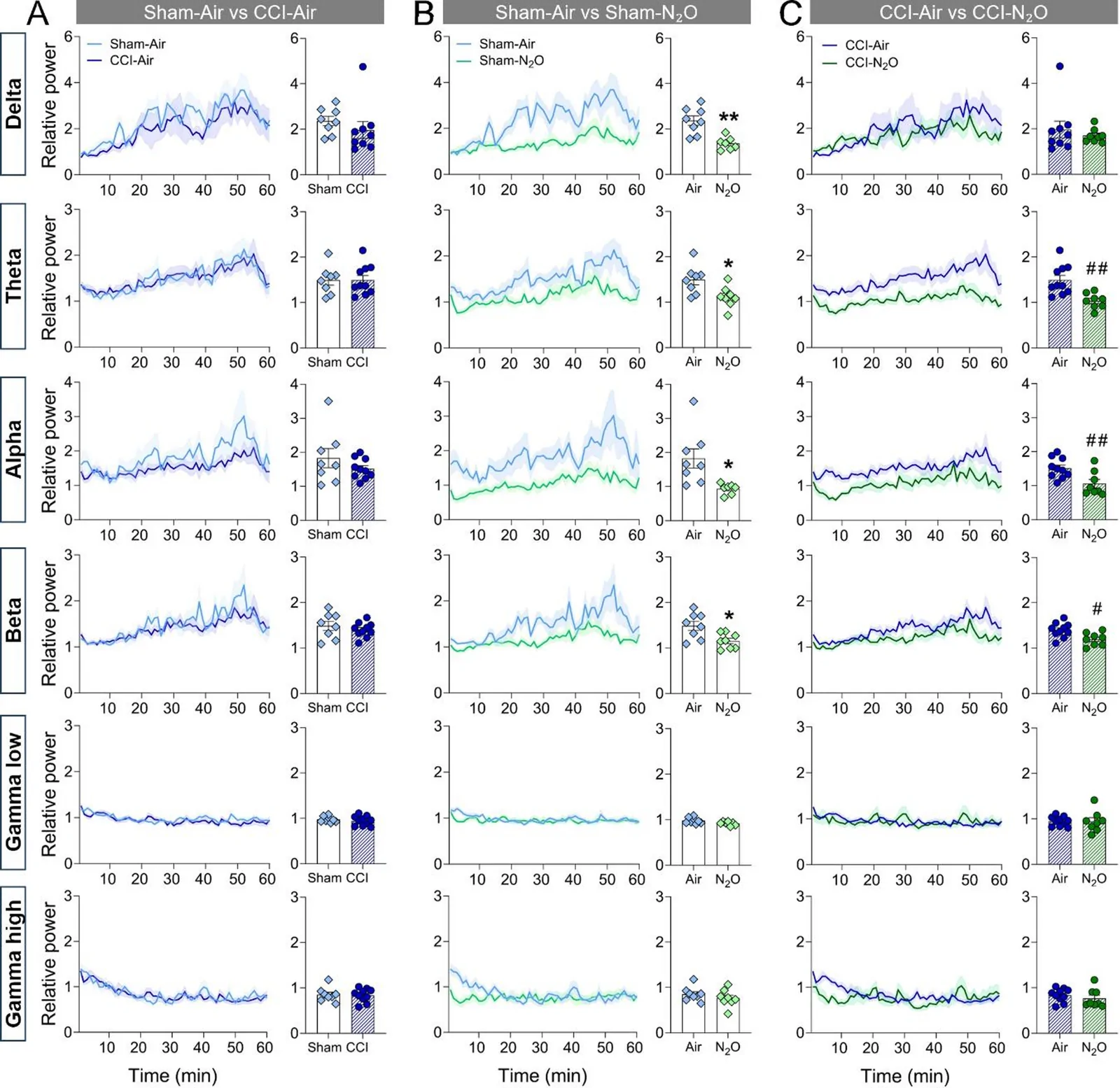
Impact of CCI model and N₂O treatment on prefrontal EEG activity during the 1-hour post-treatment period in female mice. EEG spectral power recorded across the 1-hour post-treatment period for comparisons between **(A)** Sham-Air and CCI-Air groups (*n* = 8–10), **(B)** Sham-Air and Sham-N_2_O groups (*n* = 8), and **(C)** CCI-Air and CCI-N_2_O groups (*n* = 8–10). EEG oscillatory activity was represented as time-course relative power (normalized to baseline), together with comparisons of the average total power for each frequency band: delta (1–4 Hz), theta (4–8 Hz), alpha (8–12 Hz), beta (12–30 Hz), gamma low (30–45 Hz), and gamma high (60–100 Hz). Data are presented as mean ± SEM. *p < 0.05, **p < 0.01 vs. Sham-Air; ^#^*p* < 0.05, ^##^*p* < 0.01 vs. CCI-Air; Student’s two-tailed unpaired *t* test or Mann-Whitney *U* test. Detailed statistical results are provided in Table S3

In a separate experimental group, prefrontal EEG oscillatory activity was assessed in both male (Fig. 2; Supplementary Table S2) and female (Fig. 3; Supplementary Table S3) mice during the 1-hour post-treatment period, following the same time protocol used for locomotor activity. In males, no differences were detected between Sham-Air and CCI-Air groups in any of the frequency bands (Fig. 2A). In sham male mice, N₂O treatment significantly reduced spectral power in the alpha and beta frequency bands compared with Sham-Air controls (both bands *p* < 0.01; Fig. 2B). Under the neuropathic pain condition, N₂O also reduced EEG spectral power, with CCI-N₂O mice showing significantly decreased power in the delta, theta, and beta bands, as well as in the gamma high band, compared with CCI-Air mice (delta and theta *p* < 0.05; beta *p* < 0.01, gamma high *p* < 0.05; Fig. 2C).

Prefrontal EEG oscillatory activity was similarly modulated in female mice (Fig. 3; Supplementary Table S3). As in males, no differences were detected between Sham-Air and CCI-Air groups (Fig. 3A). However, N₂O treatment induced a reduction in spectral power in sham females, with significant decreases in the delta, theta, alpha, and beta bands when comparing the Sham-Air and Sham-N₂O groups (delta *p* < 0.01; theta, alpha and beta *p* < 0.05; Fig. 3B). In CCI females, significant reductions were detected in the theta, alpha, and beta bands in the CCI-Air versus CCI-N₂O comparison (theta and alpha: *p* < 0.01; beta: *p* < 0.05; Fig. 3C).

### Effects of N₂O on nociceptive behavior and prefrontal EEG activity during sensory stimulation

**Fig. 4:**
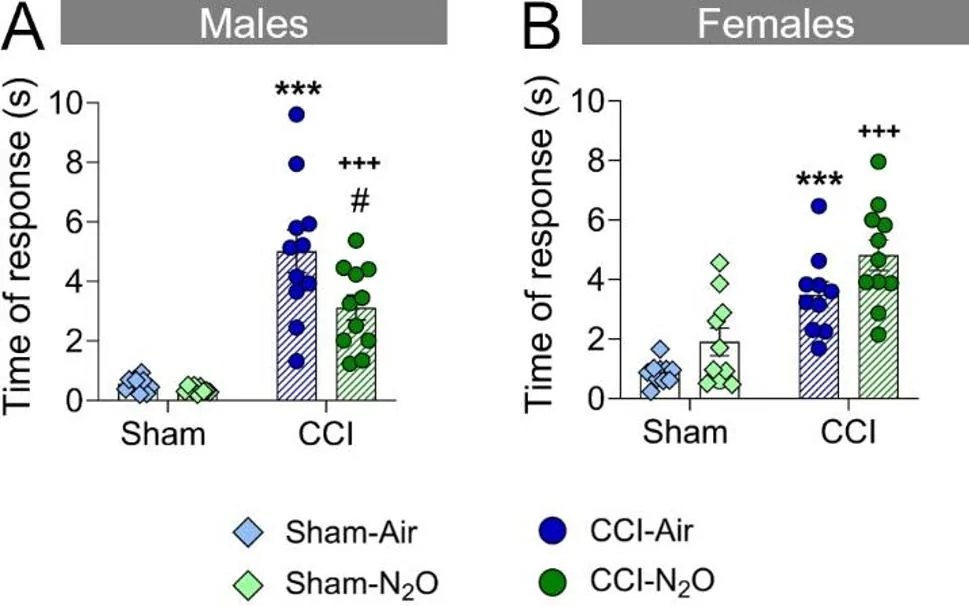
Effect of CCI and N_2_O treatment on nociceptive behavior in male and female mice. Cold hypersensitivity assessed immediately after the 1-hour post-treatment period by the acetone test, represented by time of response (in seconds) for male **(A)** and female **(B)** mice. Data are presented as mean ± SEM (*n* = 9–11 per group). ***p < 0.01 vs. Sham-Air; ^#^*p* < 0.05 vs. CCI-Air; ^+++^*p* < 0.01 vs. Sham-N₂O; 2-way ANOVA followed by Tukey’s *post hoc* test. Detailed statistical results are provided in Table S4

Immediately after the 1-hour EEG recordings, thermal hypersensitivity was assessed in male and female mice using the acetone test (Fig. 4; Supplementary Table S4).

In male mice, two-way ANOVA revealed significant main effects of model (*p* < 0.0001) and treatment (*p* < 0.05). The CCI model robustly induced cold hypersensitivity, as evidenced by the significantly increased time of response in CCI-Air compared with Sham-Air animals (*p* < 0.001; Fig. 4A), while N₂O treatment significantly reduced time of response in CCI male mice (*p* < 0.05; Fig. 4A), indicating an analgesic effect. However, N₂O was not able to fully recover the pain phenotype, such that CCI-N₂O animals still showed significantly longer response times than Sham-N₂O controls (*p* < 0.001; Fig. 4A). Notably, the analgesic effect in males was maintained for at least 6 h (*p* < 0.05) but was no longer evident 24 h after treatment in an independent group of animals (Fig. S7).

In female mice, two-way ANOVA also revealed significant main effects of model (*p* < 0.0001) and treatment (*p* < 0.01) in the acetone test. The CCI model likewise reliably induced cold hypersensitivity, as shown by CCI-Air females exhibiting longer response times than Sham-Air controls (*p* < 0.001; Fig. 4B), as well as CCI-N₂O compared with Sham-N₂O animals (*p* < 0.001; Fig. 4B). *Post hoc* comparisons between Air-treated and N_2_O-treated groups did not reach statistical significance.

**Fig. 5:**
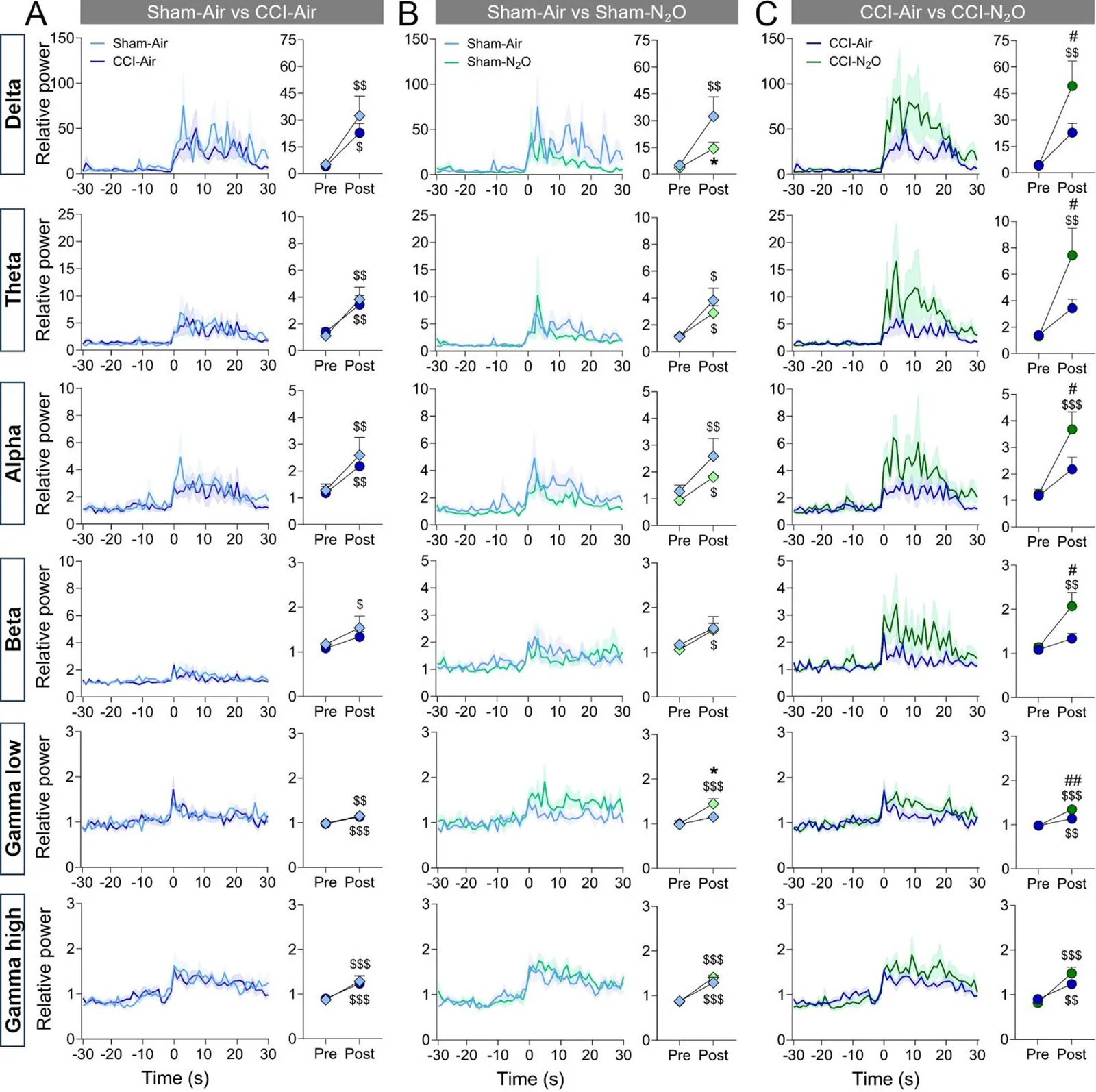
Effect of CCI model and N₂O treatment on prefrontal EEG activity during sensory stimulation in male mice. EEG spectral power recorded across a 60-second period (30s pre-acetone, and 30s post-acetone) for comparisons between **(A)** Sham-Air and CCI-Air groups (*n* = 5–7), **(B)** Sham-Air and Sham-N_2_O groups (*n* = 5–6) and **(C)** CCI-Air and CCI-N_2_O groups (*n* = 7–10). EEG oscillatory activity is represented as relative power (normalized to baseline) across the 60-second period (time zero represents the acetone application), together with comparisons of the average spectral power of 30-second Pre and 30-second Post periods for each frequency band: delta (1–4 Hz), theta (4–8 Hz), alpha (8–12 Hz), beta (12–30 Hz), gamma low (30–45 Hz), and gamma high (60–100 Hz). Data are presented as mean ± SEM. $*p* < 0.05, $$*p* < 0.01, $$$*p* < 0.001 Pre vs. Post within each group; *p < 0.05 vs. Sham-Air; #*p* < 0.05, ##*p* < 0.01 vs. CCI-Air; RM ANOVA or mixed-effects models (REML) followed by Uncorrected Fisher’s LSD *post hoc* test. Detailed statistical results are provided in Table S5

**Fig. 6:**
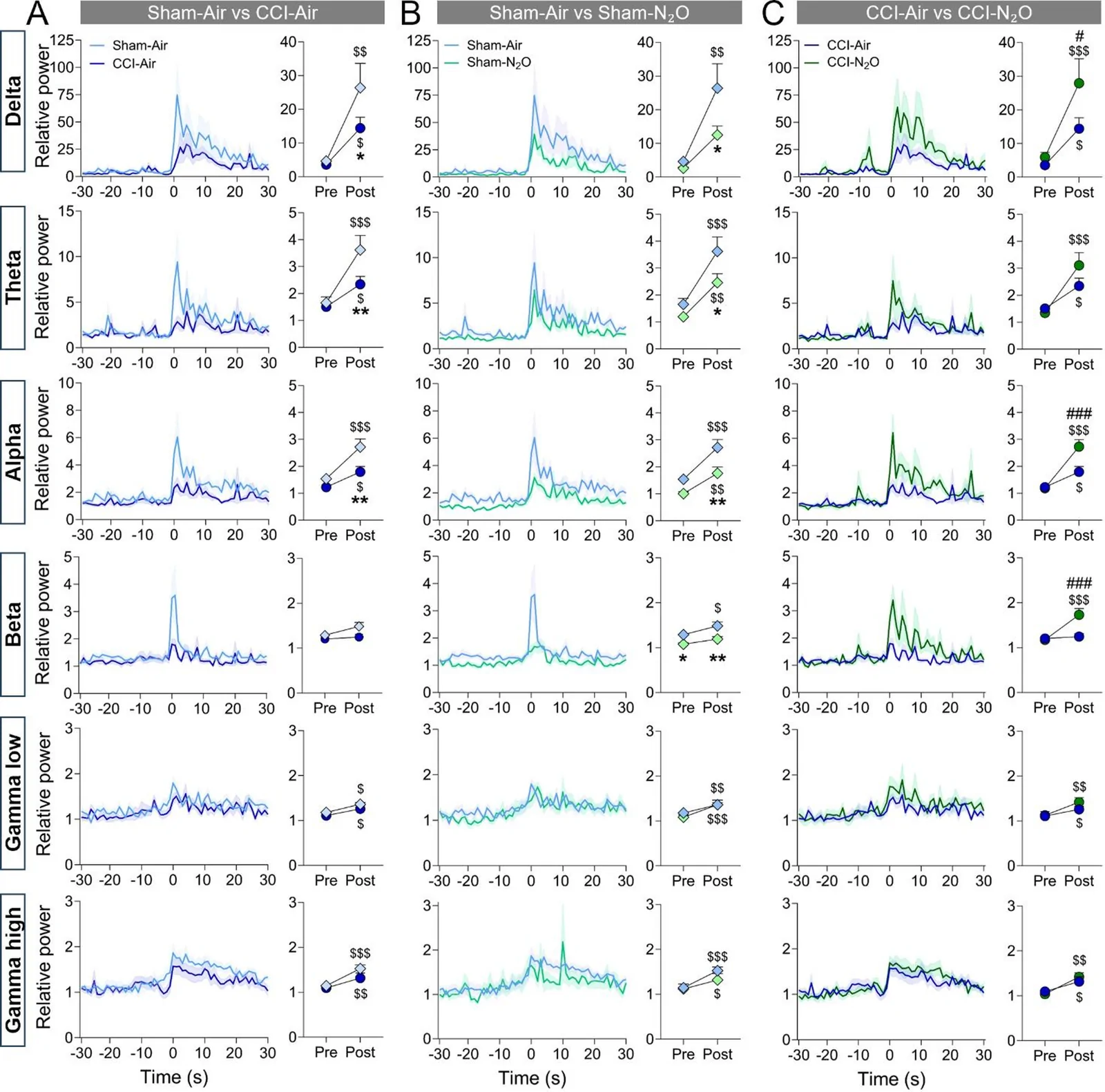
Effect of CCI model and N₂O treatment on prefrontal EEG activity during sensory stimulation in female mice. EEG spectral power recorded across a 60-second period (30s pre-acetone, and 30s post-acetone) for comparisons between **(A)** Sham-Air and CCI-Air groups (*n* = 8–10), **(B)** Sham-Air and Sham-N_2_O groups (*n* = 8) and **(C)** CCI-Air and CCI-N_2_O groups (*n* = 8–10). EEG oscillatory activity is represented as relative power (normalized to baseline) across the 60-second period (time zero represents the acetone application), together with comparisons of the average spectral power of 30-second Pre and 30-second Post periods for each frequency band: delta (1–4 Hz), theta (4–8 Hz), alpha (8–12 Hz), beta (12–30 Hz), gamma low (30–45 Hz), and gamma high (60–100 Hz). Data are presented as mean ± SEM. $*p* < 0.05, $$*p* < 0.01, $$$*p* < 0.001 Pre vs. Post within each group; *p < 0.05, **p < 0.01 vs. Sham-Air; #*p* < 0.05, ###*p* < 0.001 vs. CCI-Air; RM ANOVA or mixed-effects models (REML) followed by Uncorrected Fisher’s LSD *post hoc* test. Detailed statistical results are provided in Table S6

The acetone test was conducted alongside simultaneous EEG recordings, allowing us to assess prefrontal oscillatory activity in response to sensory stimulation following N₂O administration. A clear electrophysiological response was observed immediately after the acetone application, characterized by an abrupt general increase in spectral power in both male (Fig. 5) and female (Fig. 6), sham and CCI mice.

In males, in the Sham-Air versus CCI-Air comparison (Fig. 5A), repeated-measures ANOVA revealed a significant main effect of time (at least *p* < 0.05 across all frequency bands), but no significant main effect of model was detected (Supplementary Table S5). *Post hoc* analyses within each group identified significant differences between pre- and post-acetone intervals across all frequency bands in the Sham-Air group (delta, theta and alpha *p* < 0.01, beta *p* < 0.05, gamma low *p* < 0.01, gamma high *p* < 0.001; Fig. 5A). In the CCI-Air group, significant differences were observed across all frequency bands except beta (delta *p* < 0.05, theta and alpha *p* < 0.01, gamma low and high *p* < 0.001; Fig. 5A). *Post hoc* between-group analyses did not reach statistical significance in either the pre- or post-acetone periods.

In Sham male mice, repeated-measures ANOVA comparing Sham-Air and Sham-N₂O groups (Fig. 5B) was also significant for the main effect of time (at least *p* < 0.05 for all frequency bands), but no significant main effect of treatment was detected in any band. Nevertheless, a significant interaction between time and treatment (Time x Treatment: *p* < 0.05) was observed in the gamma low band (Supplementary Table S5). *Post hoc* analyses within each group identified significant differences between pre- and post-acetone intervals across all frequency bands, except beta and gamma low, in the Sham-Air group (delta *p* < 0.01, theta *p* < 0.05, alpha *p* < 0.01, gamma high *p* < 0.001; Fig. 5B). In the Sham-N_2_O group they were significant for all frequency bands except delta (theta, alpha and beta *p* < 0.05, gamma low and gamma high *p* < 0.001; Fig. 5B). *Post hoc* between-group analyses revealed significant differences between Sham-Air and Sham-N₂O groups in the post-acetone interval. In the delta band, the Sham-N₂O group showed decreased spectral power compared with Sham-Air (*p* < 0.05; Fig. 5B). In contrast, in the gamma low band, Sham-N₂O animals exhibited significantly higher spectral power than Sham-Air (*p* < 0.05; Fig. 5B).

Under the neuropathic pain condition **(**Fig. 5C**)**, repeated-measures ANOVA revealed a significant main effect of time (at least *p* < 0.01 across all frequency bands), but the main effect of treatment also failed to reach significance in the low-frequency bands. However, a significant interaction between time and treatment was observed for both gamma low and high bands (Time × Treatment: *p* < 0.01 and *p* < 0.05, respectively; Supplementary Table S5). *Post hoc* analyses comparing pre- versus post-acetone intervals within each group showed significant differences in the CCI-Air group only for the gamma bands (gamma low and high *p* < 0.01; Fig. 5C). In contrast, in the CCI-N_2_O group significant differences were observed across all frequency bands (delta, theta *p* < 0.01, alpha *p* < 0.001, beta *p* < 0.01, gamma low and gamma high *p* < 0.001; Fig. 5C). *Post hoc* between-group analyses revealed significant differences in the post-acetone interval across all frequency bands except gamma high, with CCI-N₂O males exhibiting higher spectral power compared to CCI-Air controls (delta, theta, alpha and beta *p* < 0.05, gamma low *p* < 0.01; Fig. 5C).

In females, in the Sham-Air versus CCI-Air comparison (Fig. 6A), repeated-measures ANOVA revealed a significant main effect of time (at least *p* < 0.01 across all frequency bands except beta). Additionally, a significant main effect of model was detected in the alpha band (Model: *p* < 0.05; Supplementary Table S6). *Post hoc* analyses within each group showed significant pre- to post-acetone differences across all frequency bands in both groups, except for beta, for which the ANOVA was not significant (Sham-Air: delta *p* < 0.01, theta and alpha *p* < 0.001, gamma low *p* < 0.05, gamma high *p* < 0.001; CCI-Air: delta, theta, alpha and gamma low *p* < 0.05, gamma high *p* < 0.01; Fig. 6A). *Post hoc* between-group analyses were significant in the post-acetone period for the low-frequency bands (delta *p* < 0.05, theta and alpha *p* < 0.01; Fig. 6A).

In sham female mice (Fig. 6B), repeated-measures ANOVA was also significant for the main effect of time (at least *p* < 0.01 for all frequency bands). In addition, N₂O induced a significant main effect of treatment in the theta, alpha, and beta bands (Treatment: *p* < 0.05 for theta and alpha; *p* < 0.01 for beta; Supplementary Table S6). *Post hoc* analyses within each group identified significant differences between pre- and post-acetone intervals across all frequency bands in the Sham-Air group (delta *p* < 0.01, theta and alpha *p* < 0.001, beta *p* < 0.05, gamma low *p* < 0.01, gamma high *p* < 0.001; Fig. 6B). In the Sham-N_2_O group they were significant across all frequency bands except delta and beta (theta and alpha *p* < 0.01, gamma low *p* < 0.001; gamma high *p* < 0.05; Fig. 6B). *Post hoc* between-group analyses revealed significant differences in the post-acetone interval across all low-frequency bands, with Sham-N₂O females exhibiting significantly lower spectral power compared to Sham-Air controls (delta and theta *p* < 0.05; alpha and beta *p* < 0.01; Fig. 6B). Moreover, the Sham-N₂O group also showed significantly lower spectral power in the beta band during the pre-acetone interval (*p* < 0.05; Fig. 6B).

Under the neuropathic pain condition (Fig. 6C), repeated-measures ANOVA revealed a significant main effect of time (at least *p* < 0.01 across all frequency bands), while the main effect of treatment was not significant for most bands, except for alpha and beta bands, which additionally showed a significant interaction between time and treatment (Time x Treatment: *p* < 0.01 for both bands; Supplementary Table S6). *Post hoc* analyses comparing pre- versus post-acetone intervals within each group showed significant differences across all frequency bands except beta in the CCI-Air group (delta, theta, alpha, gamma low and gamma high *p* < 0.05; Fig. 6C). In the CCI-N_2_O group, significant differences were observed across all frequency bands (delta, theta, alpha and beta *p* < 0.001, gamma low and gamma high *p* < 0.01; Fig. 6C). *Post hoc* between-group analyses revealed significant differences in the post-acetone interval in the delta, alpha and beta bands, with CCI-N₂O females exhibiting significantly higher spectral power compared to CCI-Air controls (delta *p* < 0.05, alpha and beta *p* < 0.001; Fig. 6C).

### Effects of N₂O treatment on depressive-like behavior

**Fig. 7:**
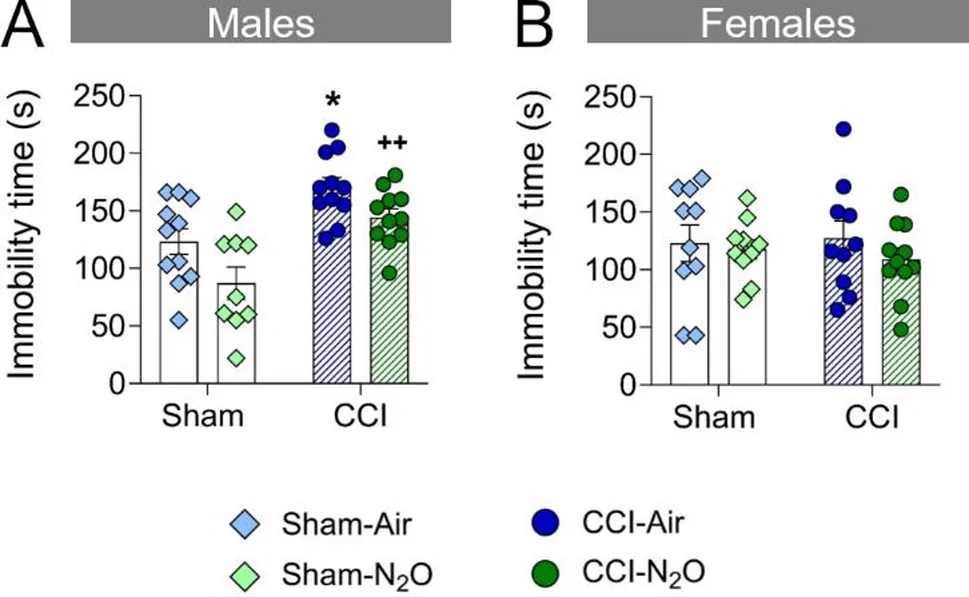
Effect of CCI and N₂O treatment on depressive-like behavior in male and female mice. Depressive-like behavior assessed 24 h after treatment administration by the tail suspension test (TST), represented by immobility time (in seconds) for male **(A)** and female **(B)** mice. Data are presented as mean ± SEM (*n* = 9–11 per group). *p < 0.05 vs. Sham-Air; ^++^*p* < 0.01 vs. Sham-N₂O; 2-way ANOVA followed by Tukey’s *post hoc* test. Detailed statistical results are provided in Table S4

Finally, 24 h after treatment administration, the TST was used to evaluate depressive-like behavior (Fig. 7; Supplementary Table S4). In male mice, two-way ANOVA revealed significant main effects of model (*p* < 0.0001) and treatment (*p* < 0.01). The CCI model induced behavioral despair, as evidenced by increased immobility time in the CCI-Air group compared with Sham-Air controls (*p* < 0.05; Fig. 7A). However, N₂O did not significantly reduce immobility time in either sham or CCI mice, despite the significant main effect of treatment in the ANOVA (Fig. 7A). Accordingly, the CCI-N₂O group still exhibited significantly longer immobility times than the Sham-N_2_O controls (Fig. 7A). In females, no significant main effects of model or treatment were observed (Fig. 7B).

## Discussion

The present study evaluated the behavioral and electrophysiological effects of N₂O (50%, 1 h) in adult male and female mice subjected to the CCI model, assessing its potential to simultaneously target nociceptive and affective symptoms. No model-dependent differences in locomotor behavior or resting prefrontal EEG activity were observed between sham and CCI animals. However, the CCI model reliably induced a pain phenotype in both male and female mice, but a depressive-like phenotype only in males. In the acetone test, stimulus application evoked a broad increase in oscillatory EEG activity across frequency bands, groups, and sexes. Notably, CCI-Air females exhibited an attenuated electrophysiological response in the low-frequency bands compared with Sham-Air controls, an effect not clearly observed in males. On the other hand, N₂O modulated behavioral and electrophysiological outcomes in a sex- and state-dependent manner. It induced a robust suppression of locomotor activity in both males and females, similarly affecting sham and CCI animals, although the effects were longer-lasting in males. At the EEG level, N₂O produced a widespread reduction in cortical oscillatory activity during the first hour post-treatment, with similar patterns across sexes but more generalized effects in females. Behaviorally, N₂O exerted analgesic effects in CCI males, whereas no significant effects were observed in females. In sham animals, N_2_O attenuated the electrophysiological response to sensory stimulation, however, in CCI mice, the post-stimulus power response was enhanced in both sexes, suggesting a restoration of prefrontal EEG activity. Finally, although a main effect of treatment was detected for males in the TST, N₂O did not significantly reduce behavioral despair in either sham or CCI mice 24 h after treatment, while no effects of model or treatment were observed in females.

Consistent with its central depressant properties, N₂O produced a marked reduction in locomotor activity across experimental conditions during the 1 h post-treatment period. In males, this effect was robust and sustained, with significant decreases in total distance traveled observed in both sham and CCI groups for approximately 35–40 min following gas withdrawal, in agreement with previous reports [24]. In females, N₂O also reduced locomotor activity; however, this effect appeared more transient, being largely restricted to the first 15–20 min post-exposure. The present data do not permit direct sex comparisons, and therefore any interpretation of sex-dependent effects should be made with caution. Notably, although N₂O effects in females were transient, air-treated female mice exhibited a more rapid decline in spontaneous exploratory behavior than males, potentially narrowing the window for detecting N₂O-induced hypolocomotion. Therefore, the apparent difference in effect duration may reflect both baseline behavioral dynamics and treatment-related effects.

At the neurophysiological level, N₂O induced a robust and wideband suppression of cortical activity during the first hour post-treatment, suggesting a broad alteration of cortical network dynamics rather than a frequency-specific modulation of neural processing. In male mice, N₂O reduced power in the alpha and beta bands under sham conditions, whereas in CCI animals this effect extended to delta, theta, and high gamma frequencies, indicating a broader disruption of ongoing network activity in the presence of chronic pain. In contrast, females exhibited a more homogeneous suppression of spectral power across delta to beta bands regardless of condition, suggesting a generalized cortical dampening that was less influenced by neuropathic state. Therefore, our results indicate that, after inhalation, N₂O elicits a consistent suppressive effect on both locomotion and cortical activity across sexes, while the temporal dynamics and spectral expression of these effects are shaped by sex and underlying physiological state. The observed behavioral and EEG suppression is consistent with the transient sedative-like state previously described following N₂O treatment [24], and may reflect a transient reduction in arousal, attentional engagement, information-processing capacity or diminished responsiveness to environmental stimuli. These post-treatment EEG changes may represent adaptive or homeostatic network responses to the alterations in cortical excitability induced by NMDA receptor antagonism. However, the interpretation of these findings is constrained by the limited and heterogenous literature examining the effects of N₂O on cortical EEG activity, particularly with respect to post-exposure dynamics. Most studies have focused on recordings obtained during inhalation and have used varying N₂O concentrations and methodological approaches, contributing to substantial variability in the reported findings [28–34]. The limited clinical evidence assessing EEG activity after exposure [35], only partially align with the available preclinical literature, which suggests that vigilance state and sleep-related processes, particularly NREM slow-wave activity, may be important determinants of the neurophysiological effects of N₂O [36, 37]. Several limitations of the present study should therefore be considered when interpreting these findings. The absence of electromyography recordings prevented sleep-stage classification and limited our ability to assess the contribution of vigilance state or sleep architecture to the observed EEG changes. In addition, EEG recordings were restricted to the first hour following N₂O exposure and did not capture the temporal evolution of neural activity during inhalation or over longer recovery periods. Differences in electrode placement, the limited inclusion of females in previous studies, and the scarcity of investigations in the context of neuropathic pain may have further contributed to discrepancies across studies. Future work combining EEG recordings during and after N₂O administration, together with extended monitoring and sleep-state assessment will help clarify the functional significance and behavioral relevance of these neurophysiological changes.

To determine whether the observed global effects translated into modulation of nociceptive processing, thermal sensitivity was assessed using the acetone test. It is important to note that acetone application constitutes a cooling stimulus that is largely innocuous under physiological conditions but becomes aversive in the context of neuropathic sensitization, where it is commonly used to assess cold allodynia [38, 39]. Consistent with this, N₂O produced significant antinociception in neuropathic males, whereas no effect was observed in sham animals, likely reflecting the limited nociceptive drive of this stimulus under baseline conditions. Although the opioid-dependent analgesic effects of N₂O during inhalation are well established [15], longer-lasting reductions in neuropathic pain hypersensitivity have also been reported in nerve-injured (CCI) male rats following a single administration [40, 41]. The attenuation of thermal hypersensitivity observed in our CCI males as early as 1 h after N₂O withdrawal may therefore reflect the early emergence of plasticity adaptations associated with NMDA receptor antagonism and the reversal of central sensitization induced by nerve injury. Supporting this interpretation, rapid molecular changes in synaptic and plasticity-related pathways have been reported within 30–60 min following N₂O exposure [24, 37]. Furthermore, analgesic effects persisted 6 h post-treatment in an independent cohort of male mice, with a similar trend observed at 24 h, findings that are consistent with the sustained antihypersensitivity effects previously described in CCI rats.

Focusing on females, this work is, to our knowledge, the first preclinical study to examine the effects of N₂O on nociception. Although statistical analyses revealed a significant main effect of treatment; *post hoc* comparisons did not reach significance. Nevertheless, the data showed an unexpected trend toward increased nociceptive sensitivity following N₂O exposure. Given the absence of significant pairwise differences, this observation should be considered hypothesis-generating rather than conclusive. Interestingly, these findings are broadly consistent with evidence indicating that neuropathic pain engages sex-dependent neuroimmune and descending modulatory mechanisms, which may differentially interact with NMDA receptor antagonism [42–44]. Indeed, the lack of analgesic efficacy in females may reflect the comparatively less robust opioid-mediated analgesia described in this sex [45–47], together with the reduced excitability of locus coeruleus neurons observed in CCI females [21]. As N₂O-induced analgesia is thought to depend on opioid-mediated activation of descending noradrenergic pathways and subsequent spinal noradrenaline release [48], these factors could collectively contribute to a less effective analgesic response in females. If substantiated in future studies, a potential pronociceptive effect of N₂O could carry important clinical implications given the widespread use of N₂O for labor analgesia and other painful procedures affecting women exclusively [49, 50]. Careful investigation will be necessary to determine whether these preclinical observations translate to humans.

Simultaneous EEG recordings during acetone stimulation further revealed that the stimulus induced an increase in spectral power across all groups, indicating that nociceptive stimulation elicited robust cortical responses [51] and arguing against a generalized suppression of neural activity or persistent sedative effects of N₂O at the time of testing. However, the modulation of these responses by N₂O diverged from behavioral outcomes. In males, N₂O reduced spectral power responses in sham animals, most prominently in the delta band, yet increased post-stimulus power across multiple frequency bands in CCI animals, despite producing clear behavioral analgesia. In females, N₂O attenuated acetone-evoked spectral increases in sham animals, whereas in CCI females it enhanced post-stimulus power across several frequency bands, mirroring the pattern observed in males but without accompanying analgesic effects. Our findings are in agreement with preclinical [52] and clinical [53] reports showing that N₂O suppresses stimulus-evoked EEG and evoked potential responses to both painful and non-painful stimuli, indicating a broad attenuation of sensory processing in baseline conditions. Importantly, both studies were conducted exclusively in male subjects, limiting insight into sex differences and comparison with our findings in females. Together, these findings demonstrate a dissociation between cortical activity and behavioral pain responses, suggesting that prefrontal EEG responses to acetone, rather than nociceptive processing per se, may reflect higher-order network processes such as emotional, motivational, attentional, or expectancy-related components of pain, which appear to be impaired (reduced) in CCI animals and restored by N_2_O. Thus, while acetone-evoked EEG changes reliably reflect the sensory salience of the stimulus, they do not directly predict analgesic efficacy under N₂O. One possible interpretation is that N₂O differentially modulates the salience assigned to sensory stimuli depending on the underlying physiological state. Specifically, N₂O may reduce the salience of sensory inputs in control animals, whereas under chronic pain conditions it may shift cortical processing toward a more responsive state that enhances the behavioral relevance of sensory stimuli. Such a shift could reflect an increased allocation of attentional resources to sensory events, independent of their perceived pain intensity, and may be related to the altered network dynamics that characterize chronic pain states. Nevertheless, the functional significance of specific oscillatory components in chronic pain remains incompletely understood, and future studies should further investigate how cortical oscillations relate not only to nociception, but also to the affective, cognitive, and learning-related dimensions of persistent pain.

In addition to nociception, depressive-like behavior was also assessed 24 h after treatment administration. Neuropathic male mice exhibited increased behavioral despair relative to sham controls, consistent with extensive evidence that chronic pain states are associated with comorbid depressive-like phenotypes in rodents [20–22, 54, 55]. However, although analysis of variance revealed a significant main effect of treatment; N₂O did not significantly reduce immobility time of either sham or CCI treated groups. Although this limits definitive conclusions, the data showed a trend toward an antidepressant-like effect 24 h after N₂O exposure. Preclinical evidence in this area remains limited and heterogeneous. Prior studies in stress-based paradigms have reported mixed results, with N₂O reversing selected behavioral deficits after single or repeated exposure, albeit not consistently across studies or depression-related behavioral assays [24, 36, 56, 57]. Notably, most preclinical studies to date have been conducted exclusively in males, further limiting generalizability of these findings. Within this context, the trend observed in the present study adds to the limited body of evidence suggesting that N₂O may produce antidepressant-like effects in male rodents, and extends this possibility to chronic pain models, although without providing definitive evidence. Notably, future studies evaluating earlier post-treatment time points may be informative, as it remains possible that the 24-hour assessment did not capture the peak behavioral effects of N₂O. Nevertheless, our findings are in line with the current available literature indicating that behavioral and network-level effects of N₂O are generally subtle and highly protocol-dependent [13], and therefore larger sample sizes and greater standardization of experimental parameters will likely be required to reliably detect these treatment-induced behavioral effects. Nonetheless, the observed pattern parallels the behavioral effects detected in nociceptive measures at 24 h—namely, a trend toward antidepressant-like and analgesic effects—raising the possibility of coordinated modulation of sensory and affective domains through shared glutamatergic mechanisms linking pain relief and affective improvement.

In contrast, no comparable behavioral effects were observed in females, in whom neither neuropathy nor N₂O significantly produced despair-like behavior or analgesia under the conditions tested. This is consistent with reports indicating that both depressive-like outcomes in neuropathic pain models and responses to glutamatergic interventions exhibit pronounced sex differences, potentially reflecting divergent engagement of limbic, neuroimmune, and monoaminergic systems [44, 58, 59]. Additionally, depressive-like phenotypes following nerve-injury have been reported to be more robust or emerge earlier in males than in females [60, 61]. Thus, the absence of an affective phenotype in our female cohort may reflect a delayed trajectory that was not captured within the present experimental window. Additionally, it is possible that female mice at this time point do not exhibit robust behavioral despair in the TST but may instead display alterations in other domains, such as anhedonia or motivational deficits. Future studies should therefore incorporate multiple behavioral assays, extended post-injury time points, and varying N₂O dosing regimens to more comprehensively define the magnitude, temporal profile, and potential sex specificity of its antidepressant effects, as well as the underlying molecular mechanisms.

### Perspectives and significance

When considered together, these behavioral and electrophysiological findings reveal a striking sex-dependent dissociation between cortical activity and behavioral outcomes. Across all groups, N₂O induced a generalized suppression of spontaneous locomotion and EEG activity after exposure, consistent with a global disruption of coordinated cortical dynamics [28, 62, 63]. This effect may reflect a transient reorganization or recalibration of pain- and affect-related cortical networks following NMDA receptor antagonism, potentially contributing to the therapeutic effects of N₂O. However, neither the spontaneous EEG activity nor the acetone-evoked EEG responses were consistently associated with behavioral improvements. In males, N₂O enhanced stimulus-evoked spectral power in CCI animals despite producing clear behavioral analgesia and a trend toward reduced despair-like behavior. In contrast, females exhibited similar electrophysiological patterns without corresponding improvements in pain or affective behaviors. Collectively, these findings indicate that cortical activity alone is insufficient to account for pain perception or its modulation and suggest that the behavioral effects of N₂O depend on the recruitment of pain-related circuits and downstream or parallel mechanisms, not directly captured by prefrontal EEG recordings, that are preferentially engaged under neuropathic conditions and differentially organized between sexes.

## Conclusion

Taken together, these findings support a model in which a single administration of 50% N₂O produces a broad modulation of cortical excitability, while its behavioral efficacy likely depends on the engagement of subcortical and descending modulatory circuits in a state- and sex-dependent manner. In males, particularly under neuropathic conditions, N₂O appears to recruit integrated networks linking nociceptive and affective processing, resulting in measurable behavioral improvements. In contrast, in females, N₂O predominantly alters cortical activity without clear evidence of effective downstream engagement of circuits required for analgesic or antidepressant-like outcomes under the conditions tested. These results have important translational implications. They suggest that electrophysiological signatures such as EEG changes, while sensitive to drug action, may not reliably predict therapeutic efficacy across sexes. More broadly, the present findings underscore the importance of incorporating sex as a critical biological variable in both preclinical and clinical studies, particularly in the development and evaluation of NMDA receptor–targeting interventions for pain and mood disorders.

## Supplementary Information

Below is the link to the electronic supplementary material.

Supplementary Material 1.

## Data Availability

The datasets used and analysed in the current study are available from the corresponding author on reasonable request.
