# Supplementary material for: Sex-specific electrophysiological and behavioral responses to nitrous oxide in a murine model of neuropathic pain
Source: Biol Sex Differ. 2026 Jul 7;17:161. doi: 10.1186/s13293-026-00945-2 (PMC13625456; doi:10.1186/s13293-026-00945-2)
Supplement: Supplementary file 1 [file 13293_2026_945_MOESM1_ESM.docx]

**Supplementary Information File for**

**Sex-specific electrophysiological and behavioral responses to nitrous oxide in a murine model of neuropathic pain**

Gemma González-Hernández^1,2,3^, Sonia Torres-Sanchez^3,4,5^, Daniel Sanchez-Morillo^5,6^, Tomi Rantamäki^1,2^ and Esther Berrocoso^3,4,5^

**List of Supplementary Material:**

**Supplementary Figures**

**Figure S1.** Effect of CCI model on prefrontal EEG activity during sensory stimulation in male mice.

**Figure S2.** Effect of N_2_O on prefrontal EEG activity during sensory stimulation in sham male mice.

**Figure S3.** Effect of N_2_O on prefrontal EEG activity during sensory stimulation in CCI male mice.

**Figure S4.** Effect of CCI model on prefrontal EEG activity during sensory stimulation in female mice.

**Figure S5.** Effect of N_2_O on prefrontal EEG activity during sensory stimulation in sham female mice.

**Figure S6.** Effect of N_2_O on prefrontal EEG activity during sensory stimulation in CCI female mice.

**Figure S7.** Effect of N_2_O treatment on nociceptive behavior in male mice across time.

**Supplementary Tables**

**Table S1.** Detailed statistical analysis applied in Figure 1.

**Table S2.** Detailed statistical analysis applied in Figure 2.

**Table S3.** Detailed statistical analysis applied in Figure 3.

**Table S4.** Detailed statistical analysis applied in Figure 4 and 7.

**Table S5.** Detailed statistical analysis applied in Figure 5.

**Table S6.** Detailed statistical analysis applied in Figure 6.

**Supplementary Figures**

**Figure S1. Effect of CCI model on prefrontal EEG activity during sensory stimulation in male mice.** EEG spectral power recorded across a 60-second period (30s pre-acetone, and 30s post-acetone) for Sham-Air vs CCI-Air groups comparison (n=5-7) for **(A)** Trial 1 **(B)** Trial 2 and **(C)** Trial 3 of the acetone test. EEG oscillatory activity is represented as relative power (normalized to baseline) across the 60-second period (time zero represents the acetone application), together with comparisons of the average spectral power of 30-second Pre and 30-second Post periods for each frequency band: delta (1-4Hz), theta (4-8Hz), alpha (8-12Hz), beta (12-30Hz), gamma low (30-45Hz), and gamma high (60-100Hz). Data are presented as mean ± SEM. $p<0.05, $$p<0.01, $$$p<0.001 Pre vs. Post within each group; *p<0.05 vs. Sham-Air; RM ANOVA or mixed-effects models (REML) followed by Uncorrected Fisher’s LSD *post hoc* test.

**Figure S2. Effect of N₂O treatment on prefrontal EEG activity during sensory stimulation in sham male mice.** EEG spectral power recorded across a 60-second period (30s pre-acetone, and 30s post-acetone) for Sham-Air vs Sham-N_2_O groups comparison (n=5-6) for **(A)** Trial 1 **(B)** Trial 2 and **(C)** Trial 3 of the acetone test. EEG oscillatory activity is represented as relative power (normalized to baseline) across the 60-second period (time zero represents the acetone application), together with comparisons of the average spectral power of 30-second Pre and 30-second Post periods for each frequency band: delta (1-4Hz), theta (4-8Hz), alpha (8-12Hz), beta (12-30Hz), gamma low (30-45Hz), and gamma high (60-100Hz). Data are presented as mean ± SEM. $p<0.05, $$p<0.01, $$$p<0.001 Pre vs. Post within each group; *p<0.05 vs. Sham-Air; RM ANOVA or mixed-effects models (REML) followed by Uncorrected Fisher’s LSD *post hoc* test.

**Figure S3. Effect of N₂O treatment on prefrontal EEG activity during sensory stimulation in CCI male mice.** EEG spectral power recorded across a 60-second period (30s pre-acetone, and 30s post-acetone) for CCI-Air vs CCI-N_2_O groups comparison (n=7-10) for **(A)** Trial 1 **(B)** Trial 2 and **(C)** Trial 3 of the acetone test. EEG oscillatory activity is represented as relative power (normalized to baseline) across the 60-second period (time zero represents the acetone application), together with comparisons of the average spectral power of 30-second Pre and 30-second Post periods for each frequency band: delta (1-4Hz), theta (4-8Hz), alpha (8-12Hz), beta (12-30Hz), gamma low (30-45Hz), and gamma high (60-100Hz). Data are presented as mean ± SEM. $p<0.05, $$p<0.01, $$$p<0.001 Pre vs. Post within each group; ^#^p<0.05, ^##^p<0.01 vs. CCI-Air; RM ANOVA or mixed-effects models (REML) followed by Uncorrected Fisher’s LSD *post hoc* test.

.

**Figure S4. Effect of CCI model on prefrontal EEG activity during sensory stimulation in female mice.** EEG spectral power recorded across a 60-second period (30s pre-acetone, and 30s post-acetone) for Sham-Air vs CCI-Air groups comparison (n=8-10) for **(A)** Trial 1 **(B)** Trial 2 and **(C)** Trial 3 of the acetone test. EEG oscillatory activity is represented as relative power (normalized to baseline) across the 60-second period (time zero represents the acetone application), together with comparisons of the average spectral power of 30-second Pre and 30-second Post periods for each frequency band: delta (1-4Hz), theta (4-8Hz), alpha (8-12Hz), beta (12-30Hz), gamma low (30-45Hz), and gamma high (60-100Hz). Data are presented as mean ± SEM. $p<0.05, $$p<0.01, $$$p<0.001 Pre vs. Post within each group; *p<0.05, **p<0.01 vs. Sham-Air; RM ANOVA or mixed-effects models (REML) followed by Uncorrected Fisher’s LSD *post hoc* test.

**Figure S5. Effect of N₂O treatment on prefrontal EEG activity during sensory stimulation in sham female mice.** EEG spectral power recorded across a 60-second period (30s pre-acetone, and 30s post-acetone) for Sham-Air vs Sham-N_2_O groups comparison (n=8) for **(A)** Trial 1 **(B)** Trial 2 and **(C)** Trial 3 of the acetone test. EEG oscillatory activity is represented as relative power (normalized to baseline) across the 60-second period (time zero represents the acetone application), together with comparisons of the average spectral power of 30-second Pre and 30-second Post periods for each frequency band: delta (1-4Hz), theta (4-8Hz), alpha (8-12Hz), beta (12-30Hz), gamma low (30-45Hz), and gamma high (60-100Hz). Data are presented as mean ± SEM. $p<0.05, $$p<0.01, $$$p<0.001 Pre vs. Post within each group; *p<0.05, **p<0.01, ***p<0.001 vs. Sham-Air; RM ANOVA or mixed-effects models (REML) followed by Uncorrected Fisher’s LSD *post hoc* test.

**Figure S6. Effect of N₂O treatment on prefrontal EEG activity during sensory stimulation in CCI female mice.** EEG spectral power recorded across a 60-second period (30s pre-acetone, and 30s post-acetone) for CCI-Air vs CCI-N_2_O groups comparison (n=8-10) for **(A)** Trial 1 **(B)** Trial 2 and **(C)** Trial 3 of the acetone test. EEG oscillatory activity is represented as relative power (normalized to baseline) across the 60-second period (time zero represents the acetone application), together with comparisons of the average spectral power of 30-second Pre and 30-second Post periods for each frequency band: delta (1-4Hz), theta (4-8Hz), alpha (8-12Hz), beta (12-30Hz), gamma low (30-45Hz), and gamma high (60-100Hz). Data are presented as mean ± SEM. $p<0.05, $$p<0.01, $$$p<0.001 Pre vs. Post within each group; ##p<0.01, ###p<0.001 vs. CCI-Air; RM ANOVA or mixed-effects models (REML) followed by Uncorrected Fisher’s LSD *post hoc* test.

**Figure S7. Effect of N₂O treatment on nociceptive behavior in male mice across time.**

Cold hypersensitivity assessed before treatment (baseline), 6h and 24h post-N₂O treatment by the acetone test, represented by time of response (in seconds) for male mice. Data are presented as mean ± SEM (n=5 per group).^#^p<0.05 vs. CCI-Air; RM-ANOVA followed by Tukey’s *post hoc* test.

**Supplementary Tables**

| **Table S1. Detailed statistical analysis applied in Figure 1.** | | | | |
| --- | --- | --- | --- | --- |
| ***Mixed effects model (REML)*** *(F_(DFn, DFd)_)* | | | | |
| **MALES Time Model Treatment Interactions** | | | | |
| **Total distance** (Fig. 1B) | **F_(5.4, 142.4)_= 24.31****** | F_(1, 28)_ = 3.20 | **F_(1, 28)_ = 92.76****** | Time x Model F_(11, 288)_ = 0.48  **Time x Treatment F_(11, 288)_ = 23.63******  Model x Treatment F_(1, 28)_ = 0.48  TimexModelxTreatment F_(11, 288)_ = 1.09 |
| **AUC** (Fig. 1C) | **F_(1.5, 39.02)_= 21.47****** | F_(1, 28)_ = 2.59 | **F_(1, 28)_ = 72.28****** | Time x Model F_(2, 54)_ = 0.76  **Time x Treatment F_(2, 54)_ = 81.41******  Model x Treatment F_(1, 28)_ = 0.08  TimexModelxTreatment F_(2, 54)_ = 2.14 |
| **FEMALES** |  |  |  |  |
| **Total distance** (Fig. 1D) | **F_(6.5, 171.1)_ = 33.55****** | F_(1, 28)_ = 0.10 | **F_(1, 28)_ = 14.90***** | **Time x Model F_(11, 290)_ = 2.16***  **Time x Treatment F_(11, 290)_ = 15.79******  Model x Treatment F_(1, 28)_ = 0.35  TimexModelxTreatment F_(11, 290)_ = 0.90 |
| **AUC** (Fig. 1E) | **F_(1.9, 51.86)_= 39.13****** | F_(1, 28)_ = 0.17 | **F_(1, 28)_ = 15.36***** | Time x Model F_(2, 54)_ = 2.78  **Time x Treatment F_(2, 54)_ = 50.84******  Model x Treatment F_(1, 28)_ = 0.26  TimexModelxTreatment F_(2, 54)_ = 1.35 |

| Table S2. Detailed statistical analysis applied in Figure 2. | | | | | |
| --- | --- | --- | --- | --- | --- |
| Group | Sample size (n) | | Shapiro-Wilk normality test (W, P value, Passed normality test?) | | Statistical test  (*t[df]* or *U*, p) |
| Prefrontal EEG activity in Sham-Air vs. CCI-Air male mice (Fig. 2A) | | | | | |
| Delta Sham-Air  Delta CCI-Air | | 8  9 | | 0.9185, 0.4179, Yes  0.9171, 0.3687, Yes | Student’s two-tailed unpaired *t* test  t (15) = 1.565, 0.1385 |
| Theta Sham-Air  Theta CCI-Air | | 8  9 | | 0.8935, 0.2523, Yes  0.9411, 0.5938, Yes | Student’s two-tailed unpaired *t* test  t (15) = 1.040, 0.3148 |
| Alpha Sham-Air  Alpha CCI-Air | | 8  9 | | 0.9932, 0.9986, Yes  0.9732, 0.9207, Yes | Student’s two-tailed unpaired *t* test  t (15) = 1.242, 0.2332 |
| Beta Sham-Air  Beta CCI-Air | | 8  9 | | 0.8344, 0.0660, Yes  0.9300, 0.4812, Yes | Student’s two-tailed unpaired *t* test  t (15) = 0.032, 0.9748 |
| Gamma Low Sham-Air  Gamma Low CCI-Air | | 8  9 | | 0.8636, 0.1304, Yes  0.9542, 0.7364, Yes | Student’s two-tailed unpaired *t* test  t (15) = 0.570, 0.5771 |
| Gamma High Sham-Air  Gamma High CCI-Air | | 8  9 | | 0.9364, 0.5762, Yes  0.8427, 0.0618, Yes | Student’s two-tailed unpaired *t* test  t (15) = 0.6339, 0.5357 |
| Prefrontal EEG activity in Sham-Air vs. Sham-N_2_O male mice (Fig. 2B) | | | | | |
| Delta Sham-Air  Delta Sham-N_2_O | | 8  9 | | 0.9185, 0.4179, Yes  0.8988, 0.2451, Yes | Student’s two-tailed unpaired *t* test  t (15) = 0.1127, 0.9118 |
| Theta Sham-Air  Theta Sham-N_2_O | | 8  9 | | 0.8935, 0.2523, Yes  0.8898, 0.1984, Yes | Student’s two-tailed unpaired *t* test  t (15) = 1.449, 0.1680 |
| Alpha Sham-Air  Alpha Sham-N_2_O | | 8  9 | | 0.9932, 0.9986, Yes  0.8952, 0.2254, Yes | Student’s two-tailed unpaired *t* test  **t (15) = 3.510, 0.0032**** |
| Beta Sham-Air  Beta Sham-N_2_O | | 8  9 | | 0.8344, 0.0660, Yes  0.9614, 0.8129, Yes | Student’s two-tailed unpaired *t* test  **t (15) = 3.164, 0.0064**** |
| Gamma Low Sham-Air  Gamma Low Sham-N_2_O | | 8  9 | | 0.8636, 0.1304, Yes  0.9490, 0.6791, Yes | Student’s two-tailed unpaired *t* test  t (15) = 1.275, 0.2217 |
| Gamma High Sham-Air  Gamma High Sham-N_2_O | | 8  9 | | 0.9364, 0.5762, Yes  0.9353, 0.5331, Yes | Student’s two-tailed unpaired *t* test  t (15) = 0.070, 0.9448 |
| Prefrontal EEG activity in CCI-Air vs. CCI-N_2_O male mice (Fig. 2C) | | | | | |
| Delta CCI-Air  Delta CCI-N_2_O | | 9  11 | | 0.9171, 0.3687, Yes  0.9121, 0.2581, Yes | Student’s two-tailed unpaired *t* test  **t (18) = 2.390, 0.0280*** |
| Theta CCI-Air  Theta CCI-N_2_O | | 9  11 | | 0.9411, 0.5938, Yes  0.9271, 0.3826, Yes | Student’s two-tailed unpaired *t* test  **t (18) = 2.780, 0.0124*** |
| Alpha CCI-Air  Alpha CCI-N_2_O | | 9  11 | | 0.9732, 0.9207, Yes  0.8904, 0.1405, Yes | Student’s two-tailed unpaired *t* test  t (18) = 1.617, 0.1233 |
| Beta CCI-Air  Beta CCI-N_2_O | | 9  11 | | 0.9300, 0.4812, Yes  0.9843, 0.9852, Yes | Student’s two-tailed unpaired *t* test  **t (18) = 2.919, 0.0092**** |
| Gamma Low CCI-Air  Gamma Low CCI-N_2_O | | 9  11 | | 0.9542, 0.7364, Yes  0.9120, 0.2574, Yes | Student’s two-tailed unpaired *t* test  t (18) = 1.333, 0.1990 |
| Gamma High CCI-Air  Gamma High CCI-N_2_O | | 9  11 | | 0.8427, 0.0618, Yes  0.9652, 0.8341, Yes | Student’s two-tailed unpaired *t* test  **t (18) = 2.372, 0.0290*** |

| Table S3. Detailed statistical analysis applied in Figure 3. | | | | | |
| --- | --- | --- | --- | --- | --- |
| Group | Sample size (n) | | Shapiro-Wilk normality test (W, P value, Passed normality test?) | | Statistical test  (*t[df]* or *U*, p) |
| Prefrontal EEG activity in Sham-Air vs. CCI-Air female mice (Fig. 3A) | | | | | |
| Delta Sham-Air  Delta CCI-Air | | 8  9* | | 0.9609, 0.8184, Yes  0.6991, 0.0014, No | Mann Whitney test  17 = 0.0745 |
| Theta Sham-Air  Theta CCI-Air | | 8  10 | | 0.9387, 0.5985, Yes  0.9124, 0.2975, Yes | Student’s two-tailed unpaired *t* test  t (16) = 0.007, 0.9948 |
| Alpha Sham-Air  Alpha CCI-Air | | 8  10 | | 0.8733, 0.1624, Yes  0.9554, 0.7318, Yes | Student’s two-tailed unpaired *t* test  t (16) = 1.152, 0.2661 |
| Beta Sham-Air  Beta CCI-Air | | 8  10 | | 0.9589, 0.8000, Yes  0.9914, 0.9983, Yes | Student’s two-tailed unpaired *t* test  t (16) = 0.838, 0.4146 |
| Gamma Low Sham-Air  Gamma Low CCI-Air | | 8  10 | | 0.9270, 0.4890, Yes  0.9502, 0.6703, Yes | Student’s two-tailed unpaired *t* test  t (16) = 0.769, 0.4534 |
| Gamma High Sham-Air  Gamma High CCI-Air | | 8  10 | | 0.9061, 0.3275, Yes  0.9488, 0.6538, Yes | Student’s two-tailed unpaired *t* test  t (16) = 0.175, 0.8631 |
| Prefrontal EEG activity in Sham-Air vs. Sham-N_2_O female mice (Fig. 3B) | | | | | |
| Delta Sham-Air  Delta Sham-N_2_O | | 8  7* | | 0.9609, 0.8184, Yes  0.9571, 0.7938, Yes | Student’s two-tailed unpaired *t* test  **t (13) = 4.153, 0.0011**** |
| Theta Sham-Air  Theta Sham-N_2_O | | 8  8 | | 0.9387, 0.5985, Yes  0.9596, 0.8064, Yes | Student’s two-tailed unpaired *t* test  **t (14) = 2.769, 0.0151*** |
| Alpha Sham-Air  Alpha Sham-N_2_O | | 8  7* | | 0.8733, 0.1624, Yes  0.9026, 0.3470, Yes | Student’s two-tailed unpaired *t* test  **t (13) = 2.919, 0.0120*** |
| Beta Sham-Air  Beta Sham-N_2_O | | 8  8 | | 0.9589, 0.8000, Yes  0.9002, 0.2900, Yes | Student’s two-tailed unpaired *t* test  **t (14) = 2.865, 0.0125*** |
| Gamma Low Sham-Air  Gamma Low Sham-N_2_O | | 8  7* | | 0.9270, 0.4890, Yes  0.9057, 0.3666, Yes | Student’s two-tailed unpaired *t* test  t (13) = 2.058, 0.0603 |
| Gamma High Sham-Air  Gamma High Sham-N_2_O | | 8  8 | | 0.9061, 0.3275, Yes  0.9559, 0.7700, Yes | Student’s two-tailed unpaired *t* test  t (14) = 0.9790, 0.3442 |
| Prefrontal EEG activity in CCI-Air vs. CCI-N_2_O female mice (Fig. 3C) | | | | | |
| Delta CCI-Air  Delta CCI-N_2_O | | 9*  8 | | 0.6991, 0.0014, No  0.8795, 0.1865, Yes | Mann Whitney test  35 = 0.9626 |
| Theta CCI-Air  Theta CCI-N_2_O | | 10  8 | | 0.9124, 0.2975, Yes  0.9704, 0.9009, Yes | Student’s two-tailed unpaired *t* test  **t (16) = 3.593, 0.0024**** |
| Alpha CCI-Air  Alpha CCI-N_2_O | | 10  8 | | 0.9554, 0.7318, Yes  0.8437, 0.0823, Yes | Student’s two-tailed unpaired *t* test  **t (16) = 2.932, 0.0098**** |
| Beta CCI-Air  Beta CCI-N_2_O | | 10  8 | | 0.9914, 0.9983, Yes  0.9641, 0.8482, Yes | Student’s two-tailed unpaired *t* test  **t (16) = 2.763, 0.0139*** |
| Gamma Low CCI-Air  Gamma Low CCI-N_2_O | | 10  8 | | 0.9502, 0.6703, Yes  0.9270, 0.4891, Yes | Student’s two-tailed unpaired *t* test  t (16) = 0.023, 0.9820 |
| Gamma High CCI-Air  Gamma High CCI-N_2_O | | 10  8 | | 0.9488, 0.6538, Yes  0.7982, 0.0274, No | Mann Whitney test  29 = 0.3599 |

*One value identified as an outlier according to Grubb’s test (α = 0.05)

| **Table S4. Detailed statistical analysis applied in Figure 4 and 7** | | | |
| --- | --- | --- | --- |
| **Nociceptive behavior – Acetone test (Fig. 4)** | | | |
| ***Two-way ANOVA*** *(F_(df, residual)_)* | | | |
|  | **Model** | **Treatment** | **Model x Treatment** |
| **Time of response - Males** | **F_(1, 38)_ = 68.27****** | **F_(1, 38)_ = 5.78*** | F_(1, 38)_ = 3.79 |
| **Time of response - Females** | **F_(1, 37)_ = 43.60****** | **F_(1, 37)_ = 7.94**** | F_(1, 37)_ = 0.11 |
| **Depressive-like behavior – TST (Fig. 7)** | | | |
| ***Two-way ANOVA*** *(F_(df, residual)_)* | | | |
|  | **Model** | **Treatment** | **Model x Treatment** |
| **Immobility time – Males** | **F_(1, 38)_ = 25.39****** | **F_(1, 38)_ = 8.97**** | F_(1, 38)_ = 0.25 |
| **Immobility time – Females** | F_(1, 37)_ = 0.03 | F_(1, 37)_ = 0.88 | F_(1, 37)_ = 0.26 |
|  |  |  |  |

| Table S5. Detailed statistical analysis applied in Figure 5 | | | | | | |
| --- | --- | --- | --- | --- | --- | --- |
| Prefrontal EEG activity during sensory stimulation in Sham-Air vs. CCI-Air male mice (Fig. 5A) | | | | | | |
| Groups | **Statistical test** | **Time** | **Model** | **Time x Model** | | **Subject** |
| Delta | RM Two-way ANOVA | **F_(1, 10)_ = 20.97**** | F_(1, 10)_ = 0.73 | F_(1, 10)_ = 0.74 | F_(10, 10)_ = 0.25 | |
| Theta | Mixed-effects model | **F_(1, 9)_ = 23.74***** | F_(1, 10)_ = 0.11 | F_(1, 9)_ = 0.11 | |  |
| Alpha | RM Two-way ANOVA | **F_(1, 10)_ = 22.40***** | F_(1, 10)_ = 0.23 | F_(1, 10)_ = 0.40 | | **F_(10, 10)_ = 4.96**** |
| Beta | Mixed-effects model | **F_(1, 9)_ = 7.39*** | F_(1, 10)_ = 0.50 | F_(1, 9)_ = 0.56 | |  |
| Gamma low | Mixed-effects model | **F_(1, 9)_ = 43.60****** | F_(1, 10)_ = 0.14 | F_(1, 9)_ = 0.41 | |  |
| Gamma high | RM Two-way ANOVA | **F_(1, 10)_ = 55.47****** | F_(1, 10)_ = 0.02 | F_(1, 10)_ = 0.60 | | **F_(10, 10)_ = 3.73*** |
| Prefrontal EEG activity during sensory stimulation in Sham-Air vs. Sham-N_2_O male mice (Fig. 5B) | | | | | | |
| Groups \| Statistical test \| Time \| Treatment \| Time x Treatment \| Subject | | | | | | |
| Delta | Mixed-effects model | **F_(1, 8)_ = 13.52**** | F_(1, 9)_ = 2.71 | F_(1, 8)_ = 2.60 | |  |
| Theta | Mixed-effects model | **F_(1, 8)_ = 17.29**** | F_(1, 9)_ = 0.74 | F_(1, 8)_ = 0.77 | |  |
| Alpha | Mixed-effects model | **F_(1, 8)_ = 20.05**** | F_(1, 9)_ = 2.00 | F_(1, 8)_ = 0.81 | |  |
| Beta | RM Two-way ANOVA | **F_(1, 9)_ = 8.73*** | F_(1, 9)_ = 0.19 | F_(1, 9)_ = 0.07 | | F_(9, 9)_ = 1.55 |
| Gamma low | Mixed-effects model | **F_(1, 8)_ = 32.89***** | F_(1, 9)_ = 2.61 | **F_(1, 8)_ = 5.88*** | |  |
| Gamma high | RM Two-way ANOVA | **F_(1, 8)_ = 104.5****** | F_(1, 8)_ = 0.31 | F_(1, 8)_ = 1.85 | | **F_(8, 8)_ = 4.56*** |
| Prefrontal EEG activity during sensory stimulation in CCI-Air vs. CCI-N_2_O male mice (Fig. 5C) | | | | | | |
| Groups \| Statistical test \| Time \| Treatment \| Time x Treatment \| Subject | | | | | | |
| Delta | Mixed-effects model | **F_(1, 14)_ = 12.88**** | F_(1, 15)_ = 2.16 | F_(1, 14)_ = 2.15 | |  |
| Theta | Mixed-effects model | **F_(1, 14)_ = 10.09**** | F_(1, 15)_ = 2.16 | F_(1, 14)_ = 2.54 | |  |
| Alpha | RM Two-way ANOVA | **F_(1, 15)_ = 20.06***** | F_(1, 15)_ = 2.27 | F_(1, 15)_ = 3.51 | | F_(15, 15)_ = 1.84 |
| Beta | Mixed-effects model | **F_(1, 14)_ = 9.46**** | F_(1, 15)_ = 3.03 | F_(1, 14)_ = 3.34 | |  |
| Gamma low | Mixed-effects model | **F_(1, 14)_ = 94.36****** | F_(1, 15)_ = 2.87 | **F_(1, 14)_ = 15.85**** | |  |
| Gamma high | Mixed-effects model | **F_(1, 14)_ = 55.63****** | F_(1, 15)_ = 0.68 | **F_(1, 14)_ = 4.82*** | |  |

| Table S6. Detailed statistical analysis applied in Figure 6 | | | | | | |
| --- | --- | --- | --- | --- | --- | --- |
| Prefrontal EEG activity during sensory stimulation in Sham-Air vs. CCI-Air female mice (Fig. 6A) | | | | | | |
| Groups | **Statistical test** | **Time** | **Model** | **Time x Model** | | **Subject** |
| Delta | Mixed-effects model | **F_(1, 14)_ = 19.16***** | F_(1, 16)_ = 2.49 | F_(1, 14)_ = 2.10 |  | |
| Theta | Mixed-effects model | **F_(1, 15)_ = 24.28***** | F_(1, 16)_ = 4.03 | F_(1, 15)_ = 3.73 | |  |
| Alpha | RM Two-way ANOVA | **F_(1, 15)_ = 32.74****** | **F_(1, 15)_ = 6.73*** | F_(1, 15)_ = 4.05 | | **F_(15, 15)_ = 2.46*** |
| Beta | Mixed-effects model | F_(1, 15)_ = 4.37 | F_(1, 16)_ = 3.50 | F_(1, 15)_ = 1.38 | |  |
| Gamma low | RM Two-way ANOVA | **F_(1, 16)_ = 14.48**** | F_(1, 16)_ = 1.67 | F_(1, 16)_ = 0.16 | | F_(16, 16)_ = 2.31 |
| Gamma high | Mixed-effects model | **F_(1, 15)_ = 36.10****** | F_(1, 16)_ = 1.06 | F_(1, 15)_ = 1.88 | |  |
| Prefrontal EEG activity during sensory stimulation in Sham-Air vs. Sham-N_2_O female mice (Fig. 6B) | | | | | | |
| Groups \| Statistical test \| Time \| Treatment \| Time x Treatment \| Subject | | | | | | |
| Delta | Mixed-effects model | **F_(1, 12)_ = 15.46**** | F_(1, 14)_ = 3.35 | F_(1, 12)_ = 2.23 | |  |
| Theta | RM Two-way ANOVA | **F_(1, 14)_ = 29.50****** | **F_(1, 14)_ = 4.61*** | F_(1, 14)_ = 1.36 | | F_(14, 14)_ = 1.61 |
| Alpha | RM Two-way ANOVA | **F_(1, 14)_ = 37.95****** | **F_(1, 14)_ = 8.65*** | F_(1, 14)_ = 1.84 | | F_(14, 14)_ = 2.63 |
| Beta | Mixed-effects model | **F_(1, 13)_ = 9.77**** | **F_(1, 14)_ = 11.77**** | F_(1, 13)_ = 0.60 | |  |
| Gamma low | RM Two-way ANOVA | **F_(1, 14)_ = 28.54***** | F_(1, 14)_ = 0.29 | F_(1, 14)_ = 1.17 | | **F_(14, 14)_ = 4.00**** |
| Gamma high | RM Two-way ANOVA | **F_(1, 13)_ = 21.26***** | F_(1, 13)_ = 0.69 | F_(1, 13)_ = 2.03 | | **F_(13, 13)_ = 4.97**** |
| Prefrontal EEG activity during sensory stimulation in CCI-Air vs. CCI-N_2_O female mice (Fig. 6C) | | | | | | |
| Groups \| Statistical test \| Time \| Treatment \| Time x Treatment \| Subject | | | | | | |
| Delta | Mixed-effects model | **F_(1, 15)_ = 24.49***** | F_(1, 16)_ = 3.35 | F_(1, 15)_ = 2.75 | |  |
| Theta | Mixed-effects model | **F_(1, 14)_ = 25.35***** | F_(1, 16)_ = 1.08 | F_(1, 14)_ = 3.17 | |  |
| Alpha | Mixed-effects model | **F_(1, 14)_ = 47.03****** | **F_(1, 15)_ = 4.99*** | **F_(1, 14)_ = 9.90**** | |  |
| Beta | Mixed-effects model | **F_(1, 14)_ = 14.23**** | **F_(1, 16)_ = 5.67*** | **F_(1, 14)_ = 10.02**** | |  |
| Gamma low | RM Two-way ANOVA | **F_(1, 16)_ = 19.67***** | F_(1, 16)_ = 0.88 | F_(1, 16)_ = 2.08 | | **F_(16, 16)_ = 3.64**** |
| Gamma high | Mixed-effects model | **F_(1, 14)_ = 20.67***** | F_(1, 16)_ = 0.07 | F_(1, 14)_ = 0.97 | |  |
